# PARP7 and aryl hydrocarbon receptor differentially regulate mammary cancer cell proliferation and STING-induced type I interferon signalling

**DOI:** 10.1007/s13402-025-01150-w

**Published:** 2025-12-23

**Authors:** Ninni E. Olafsen, Samaneh S. Åhrling, Marit Rasmussen, Linnea A. M. Erlingsson, Emma N. Granly, Akinori Takaoka, Jason Matthews

**Affiliations:** 1 Department of Nutrition, Institute of Basic Medical Sciences, Faculty of Medicine, University of Oslo, Oslo, Norway; 2 Division of Signalling in Cancer and Immunology, Institute for Genetic Medicine, Hokkaido University, , Sapporo, Japan; 3 Department of Pharmacology and Toxicology, University of Toronto, Toronto, ON Canada

**Keywords:** PARP7, Aryl hydrocarbon receptor, Type I interferon signalling, Proliferation, Breast cancer, RBN2397

## Abstract

**Purpose:**

PARP7 is a negative regulator of type I interferon (IFN-I) and aryl hydrocarbon receptor (AHR) signalling and has important roles in cell proliferation and antitumor immunity. Recently, several cancer cell lines have been reported to be sensitive to the antiproliferative effect of PARP7 inhibition by RBN2397; however, the roles of AHR and IFN-I signalling in this effect are not fully understood.

**Methods:**

Murine mammary cancer cells were treated with AHR ligands, RBN2397 and with the stimulator of interferon genes (STING) agonist, DMXAA. The impact of ligand treatments on AHR and IFN-I signalling and cell proliferation was determined.

**Results:**

RBN2397 enhanced AHR ligand signalling and STING-induced IFN-I responses in both cell lines. Py8119 but not Py230, 4T1 or EO771 cells were sensitive to the antiproliferative effects of RBN2397. In agreement with FOS-related antigen 1 (FOSL1) being required for sensitivity to RBN2397, Py8119 but not Py230 cells expressed FOSL1. However, RBN2397 insensitive 4T1 and EO771 cell lines also expressed FOSL1, suggesting that the role of FOSL1 in RBN2397-mediated growth inhibition exhibits cell line specificity. In Py8119 cells, RBN2397 induced apoptosis which was independent of AHR ligand treatment and DMXAA-induced STING activation. Although Py230 cells were resistant to the antiproliferative effects RBN2397 alone, combined treatment of DMXAA with RBN2397 reduced their proliferation, which was further reduced by AHR loss or its inhibition.

**Conclusion:**

These findings highlight the complexity of the interplay among PARP7, AHR and STING-induced IFN signalling in regulating cancer cell proliferation but also suggest that for some cell lines STING activation might increase their sensitivity to the anti-proliferative effects of RBN2397.

**Supplementary Information:**

The online version contains supplementary material available at 10.1007/s13402-025-01150-w.

## Introduction

ADP-ribosyltransferases (ARTs) are a family of enzymes that use nicotinamide adenine dinucleotide (NAD^+^) as a substrate to transfer one molecule of ADP-ribose or several ADP-ribose moieties to specific amino acid residues on themselves and on target proteins, referred to as mono-ARTs and poly-ARTs, respectively [1, 2]. The diphtheria toxin-like members of the ART family (referred to as ARTDs) includes 17 different proteins that regulate a variety of cellular and pathological responses [2]. The most studied member is the poly-ART, PARP1, for its roles in DNA repair, apoptosis, gene regulation and cancer [3, 4]. More recently, PARP7, a mono-ART also known as TCDD-inducible poly-ADP-ribose polymerase (TIPARP), has emerged as a critical regulator of tumour progression by promoting cancer cell growth and suppressing antitumor immunity [5].

PARP7 was first identified as an aryl hydrocarbon receptor (AHR) target gene [6]. AHR is a ligand-activated transcription factor long studied for its role as an environmental sensor that coordinates different cellular responses in a ligand-dependent manner. In the absence of ligand, AHR resides in the cytoplasm as part of a multi-protein chaperone complex. Upon ligand binding, AHR undergoes conformational changes that promote its nuclear translocation. Nuclear AHR dimerizes with AHR nuclear translocator (ARNT) to regulate the expression of its target genes, including cytochrome P450 1A1 (CYP1A1), CYP1B1 and PARP7 [7–9]. PARP7 was later shown to function as part of a negative feedback loop to repress AHR activity in a mechanism that requires its catalytic activity [10–12]. PARP7 also ADP-ribosylates and differentially modulates estrogen receptor (NR3A1), liver X receptors (NR1H2/H3) and androgen receptor (NR3C4) activities [13–15]. Additional studies revealed that PARP7 preferentially ADP-ribosylates cysteine but also glutamate residues on several proteins involved in RNA processing, cytokine signalling and effector and innate immune responses [16–18].

PARP7’s modulation of immune responses through repressing type I interferon (IFN-I) signalling has led to increased interest in targeting this enzyme for cancer treatment [19]. Nucleic acids released from damaged cells are recognized by pattern recognition receptors (PRRs) and elicit an immune response resulting in increased IFN-I signalling. Increases in cytosolic DNA from mitochondrial stress, viral and bacterial infections or damaged cells activate the cyclic GMP-AMP synthase (cGAS)-stimulator of interferon genes (STING) and TANK-binding kinase 1 (TBK1) pathway [20]. STING agonists like 5,6-dimethylxanthenone-4-acetic acid (DMXAA) and ADU-S100 have been developed to stimulate IFN-I signalling downstream of cGAS [21]. Phosphorylated and activated TBK1 subsequently stimulates interferon regulatory factor 3 (IRF3) increasing the expression of IFNα/β levels that bind to IFN receptors (IFNAR1 and 2) resulting in activation of janus activated kinase (JAK)-signal transducer and activator of transcription (STAT) pathway and other inflammatory cytokines to increase the expression of several IFN-stimulated genes (ISGs), such as the chemokine, CXCL10. Increased IFN-I signalling improves antitumour immune responses and can also directly reduce cancer cell proliferation by inducing apoptosis and autophagy [22–24].

PARP7 inhibition with RBN2397 (also known as atamparib) results in increased immune cell infiltration and decreased tumour growth that was dependent on IFN-I signalling and increased immune cell infiltration [25–27]. In addition, RBN2397 has been reported to reduce cancer cell proliferation through a cell-autonomous manner [18, 28]. CRISPR-screens revealed that loss of *AHR* and *PARP7* protect against the antiproliferative effects of RBN2397 [25, 29], while RBN2397 in combination with AHR activation synergistically increases sensitivity to RBN2397 across several cancer cell lines [30].

AHR has also been shown to repress IFN-I independently of PARP7 by inducing the proteolytic degradation of STING, and pharmacologic inhibition of AHR using BAY2416964 restored STING activity in breast cancer cell lines [31, 32]. Recently, the antiproliferative effect of RBN2397 has also been shown to occur via IFN-I induced apoptosis by regulation of FOS-related antigen 1 (FOSL1; also known as FRA1) levels [18]. In the absence of its inhibition, PARP7 ADP-ribosylates and stabilizes FOSL1 which inhibits IRF1 and IRF3 activity, thereby preventing IFN-I induced apoptosis in lung and breast cancer cell lines. However, the sensitivity of prostate cancer cells to RBN2397 is reported to be independent of IFN-I signalling [28].

To explore how AHR and IFN-I signalling together affect PARP7 inhibition on cancer cell proliferation, we used Py8119 and Py230 murine breast cancer cell lines derived from the MMTV-PyMT mouse model [33]. We identify Py8119 cells as sensitive to the anti-proliferative effects of RBN2397 by increased apoptosis that was AHR- independent and unaffected by STING-induced IFN-I signalling. Py230 cells on the other hand were sensitized to the anti-proliferative effects of RBN2397 after simultaneous STING-activation resulting increased autophagy.

## Methods

### Chemicals and reagents

Dimethyl sulfoxide (DMSO), 5F-203 and paclitaxel was purchased from Merck (Darmstadt, Germany), 6-formylindolo(3,2-b)carbazole (FICZ) from SelleckChem (Houston, TX, USA), RBN2397, BAY2416964 and tapinarof from MedChemExpress (Monmouth Junction, NJ, USA), and DMXAA and MRT67307 from Invivogen (San Diego, CA, USA). All chemicals were stored according to the manufacturer’s instructions.

### Cell lines and cell culturing

Py8119 (CRL-3278), Py230 (CRL-3279), 4T1 (CRL-2539) and EO771 (CRL-3461) cells were purchased from ATCC (Manassas, VA, USA). Both cell lines were cultured in RPMI medium (1.0 g/L glucose; Merck) supplemented with 10% *v/v* heat-inactivated fetal bovine serum (FBS; Merck), 1% *v/v* penicillin-streptomycin (Merck) and 1% *v/v* L-glutamine (Merck), and sub-cultured when they reached 80% confluency.

### Generation of AhrKO cell lines

Py8119 and Py230 *Ahr* knockout (AhrKO) cell line clones were generated using CRISPR/Cas9 as previously described [34]. Briefly, the guide oligos 5’-AAACTCTAAGCGACACAGAGACCGC-3’ and reverse 5’-CACCGCGGTCTCTGTGTCGCTTAGA-3’ containing gRNA sequencing targeting exon 2 of *Ahr* were annealed and ligated into pSpCas9(BB)-2 A-Puro (PX459) plasmid (Addgene, Watertown, MA, USA; plasmid #62988). Cell lines were transfected with 4 µg plasmid, selected with puromycin, diluted to single cell clones and expanded. After screening for AHR activity, the presence of indels and frame shift mutations were confirmed by DNA sequencing of the target site of the *Ahr* gene from the independent clones using the primers forward 5´-TGTTTCGTCGGTAGAGCAGT-3´ and reverse 5´-AGTCCTAGCCCCAATCAGTCT-3´. Py8119 and Py230 cells transfected with empty PX459 vector and exposed to puromycin were expanded and used as control cells. They are referred to as Py8119^Cas9^ and Py230^Cas9^ cells.

### Real time quantitative PCR (RT-qPCR)

Cells were plated at a density of 1 × 10^5^ cells/well in 24-well plates and treated with test chemicals the following day. Total RNA was isolated using the Aurum™ Total RNA isolation kit (BioRad, Hercules, CA, USA), and reverse transcribed to cDNA using High-Capacity cDNA Reverse Transcription kit (Thermo Fisher Scientific, Waltham, MA, USA) according to the manufacturer’s instructions. SsoAdvanced Universal SYBR^®^ Green Supermix (BioRad) was used for RT-qPCR with the following primers: *Tbp* forward 5´-GCACAGGAGCCAAGAGTGAA-3´, reverse 5´-TAGCTGGGAAGCCCAACTTC-3´, *Cyp1a1* forward 5´-CGTTATGACCATGATGACCAAGA-3´, reverse 5´-TCCCCAAACTCATTGCTCAGAT-3´, *Cyp1b1* forward 5´-CCAGATCCCGCTGCTCTACA-3´, reverse 5´-TGGACTGTCTGCACTAAGGCTG-3´, *Parp7* forward 5´-AAAACCCCTGGAAATCAACC-3´, reverse 5´-GAATCTGCCACTGTCCCACT-3´, *Ifnb* forward 5´-TGGGAGATGTCCTCAACTGC-3´, reverse 5´-CCAGGAGTAGCTGTTGTACT-3´, *Cxcl10* forward 5´-CCAAGTGCTGCCGTCATTTTC-3´, reverse 5´-GGCTCGCAGGGATGATTTCAA-3´, *Fosl1* forward 5´-CCGAAGAAAGGAGCTGACAGAC-3´, reverse 5´-CTCAAGGCGTTCCTTCTGCTTC-3´.

### Western blotting

Cells were plated at 1.5 × 10^5^ cells/well in 6-well plates and treated with test compounds the next day. The cells were harvested using 95 °C warm 10 mM Tris-HCl buffer with 1 mM EDTA and 1% SDS, pH of 8.0, prior to sonication at low intensity for 2 × 30 s on/off using a Bioruptor (Diagenode, Denville, NJ, USA). Nuclear and cytosolic cell fractions were harvested using the NE-PER™ Nuclear and Cytoplasmic Reagents kit (Thermo Fisher Scientific) according to manufacturer’s instructions. Protein concentration was determined using BCA assays (Thermo Fisher Scientific). Protein samples were heated for 5 min at 95 °C in Laemmli sample buffer (BioRad) before being separated on a 4–20% SDS-PAGE gel (BioRad) and transferred to polyvinylidene fluoride membranes (PVDF; Merck). Blocking and dilution of primary antibodies were done with 5% skim milk in TBS-T (Tris, NaCl and 0.1% Tween20) except for all phospho-antibodies which was done with 5% bovine serum albumin in TBS-T. Antibodies used were: anti-AHR (Enzo Life Sciences, Farmingdale, NY, USA; bml-sa210-0100), anti-ACTB (Merck; AC-74), anti-Lamin A/C (Cell Signalling Technology, Danvers, MA, USA; 2032), anti-α-tubulin (Merck; T5168), anti-cGAS (Thermo Fisher Scientific; 10H1L5), anti-pSTING (S365) (Cell Signalling Technology; D8F4W), anti-STING (Cell Signalling Technology; D2P2F), anti-pTBK1 (S172) (Cell Signalling Technology; D52C2), anti-TBK1 (Cell Signalling Technology; E9H5S), anti-pIRF3 (S396) (Cell Signalling Technology; D601M), anti-IRF3 (Cell Signalling Technology; D83B9), anti-IFNAR1 (Biolegend, San Diego, CA, USA), anti-pSTAT1 (Y701) (Cell Signalling Technology; D4A7), anti-STAT1 (Cell Signalling Technology; #9172), anti-STAT2 (Cell Signalling Technology; D9J7L), anti-IRF9 (Cell Signalling Technology; D9I5H), anti-pFOSL1 (S265) (Cell Signalling Technology; 5841S), anti-FOSL1 (C-12) (Santa Cruz Biotechnology, Dallas, TX, USA; sc-28310), anti-CASP3 (Cell Signalling Technology; 9662S), anti-cleaved CASP3 (Cell Signalling Technology; 9661S), anti-PARP1 (Cell Signalling Technology; 9542S), anti-LC3B (Cell Signalling Technology; 2775S), anti-γH2AX (Cell Signalling Technology; 9718S). A lab generated anti-PARP7 antibody was used as previously described [12, 15]. Membranes were incubated with primary antibodies O/N at 4 °C and after washing the membranes with TBS-T, the appropriate secondary antibodies for 1 h at room temperature. Following washing with TBS-T, protein bands were visualized using SuperSignal™ West Pico PLUS Chemiluminescent Substrate, SuperSignal™ West Dura Extended Duration Substrate or SuperSignal™ West Atto Ultimate Sensitivity Substrate (Thermo Fisher Scientific).

### Cell proliferation assays using IncuCyte and cellTiter-Glo^®^ luminescent Cell viability assay

For IncuCyte assays, cells were plated in 96-well plates at a density of 2.5 × 10^3^ cells/well in four technical replicates for each biological replicate. The following morning, the media was changed with 150 µl media containing compounds. The plates were placed in an IncuCyte S3 instrument (Sartorius, Göttingen, Germany) set to measure confluency every 6 h for up to 132 h or until 100% confluency was reached. Percentage confluency was calculated by the IncuCyte software by masking the cell outline at baseline, and further measurement of the percentage of the total area of each well that were occupied by the cells. For CellTiter-Glo^®^ assays, cells were plated in opaque 96-well plates at a density of 5 × 10^2^ cells/well in four technical replicates for each biological replicate. The baseline was measured 6 h after plating. The following day the media was changed for 150 µl media containing test compounds and incubated 7 days. Baseline and endpoint measurement of live cells were done using CellTiter-Glo^®^ Luminescent Cell Viability Assay (Promega, Madison, WI, USA) by aspirating the media and adding 50 µl of CellTiter-Glo^®^ Reagent mixed with RPMI media in a 1:1 ratio. Luminescence was measured using Synergy™ H1 Multi-Mode Microplate Reader (BioTek, Winooski, VT, USA).

### Enzyme linked immunosorbent assay (ELISA)

Cells were plated at a density of 1 × 10^5^ cells/well in 24-well plates and treated with test chemicals the following day. An aliquot of cell culture media was stored at -20 °C. ELISAs for detecting IFNB and CXCL10 were done using the mouse IFN-beta DuoSet (R&D Systems, Minneapolis, MN, USA) and the mouse CXCL10/IP-10/CRG-2 DuoSet ELISA kits (R&D Systems), respectively. Assays were done according to the manufacturer’s instructions and the absorbance at 450 nm and 570 nm was measured using a Synergy™ H1 Multi-Mode Microplate Reader (BioTek).

### Chromatin Immunoprecipitation (ChIP) assays

Cells were plated in 10 cm dishes at a density of 1.5 × 10^5^ cells per mL in a total volume of 10 mL. Cells were treated 48 h later with DMSO, 10 nM FICZ, 100 nM RBN2397 or 10 nM FICZ + 100 nM RBN2397 for one hour. 1% formaldehyde was then added, and cells were placed on a shaker for 10 min. Cross-linking was quenched by adding 125 mM glycine, and cells were washed with phosphate-buffered saline (PBS), harvested, and resuspended in lysis buffer (50 mM Tris-HCl [pH 8.0], 150 mM NaCl, 1 mM EDTA, 1% Triton X-100, 0.1% Na-deoxycholate) containing protease inhibitors (Roche, Mannheim, Germany). Cells extracts were sonicated using a bioruptor at high intensity for 15 cycles of 30s on/30s off. The soluble chromatin was collected by centrifugation, and an aliquot of the chromatin was saved as the input fraction. The supernatants were incubated with 20 µl of protein A/G Dynabeads (Thermo Fisher Scientific) under gentle agitation for 2 h at 4°C. The supernatant was transferred to a new microcentrifuge tube, and ChIP assays were performed using a negative control (no antibody) or 3 µg of anti-AHR (Enzo Life Sciences, Farmingdale, NY USA; SA-210) per immunoprecipitation and incubated overnight at 4°C. Protein A/G Dynabeads (Thermo Fisher Scientific) was then added and incubated for 1.5 h. The pellets were successively washed for 5 min in 1 ml of buffer 1 (20 mM Tris-HCl [pH 8.0], 150 mM NaCl, 2 mM EDTA, 1% Triton X-100, 0.1% sodium dodecyl sulfate [SDS]), 1 ml of buffer 2 (20 mM Tris-HCl [pH 8.0], 500 mM NaCl, 2 mM EDTA, 1% Triton X-100, 0.1% SDS), and 1 ml of LiCl buffer (20 mM Tris-HCl [pH 8.0], 250 mM LiCl, 1 mM EDTA, 1% NP-40, 1% Na-deoxycholate). Protein-DNA complexes were eluted in 110 µl of elution buffer (PBS containing 1% SDS) for 30 min, and the cross-links were reversed by overnight incubation at 65°C. DNA was purified using a PCR purification kit (QIAGEN) and eluted in 35 µl. One µL from each sample and the input samples were analysed by RT-qPCR. The primers used for *Cyp1a1* were: forward 5’-TTTGCTTCCTCACAGGGTGT-3’ and reverse 5’-AGGGTCACTTTGTTCCGAGA-3’.

### Statistical analyses

All data are presented as mean ± standard error of the mean (S.E.M.) unless otherwise specified. Statistical analyses were done using GraphPad Prism 10.2.0 (GraphPad Software, Boston, MA, USA). Significant differences were determined by unpaired student’s *t*-test, one-way analysis of variance (ANOVA), area under the curve, or non-linear fit log(inhibitor) vs. response variable slope (four parameters) when appropriate with *p* < 0.05.

## Results

### Py8119 and Py230 cells express functional AHR and IFN-I signalling pathways

Several cancer cell lines have been reported to be sensitive to the antiproliferative effects of RBN2397 [18, 25, 28]. CRISPR screens have identified AHR to be an important determinant of sensitivity to RBN2397 [25, 29], and more recently IFN-I signalling through manipulation of FOSL1 levels has been reported to also play an important role [18]. However, whether AHR affects IFN-I signalling-dependent sensitivity to RBN2397 and vice versa is unclear.

**Fig. 1:**
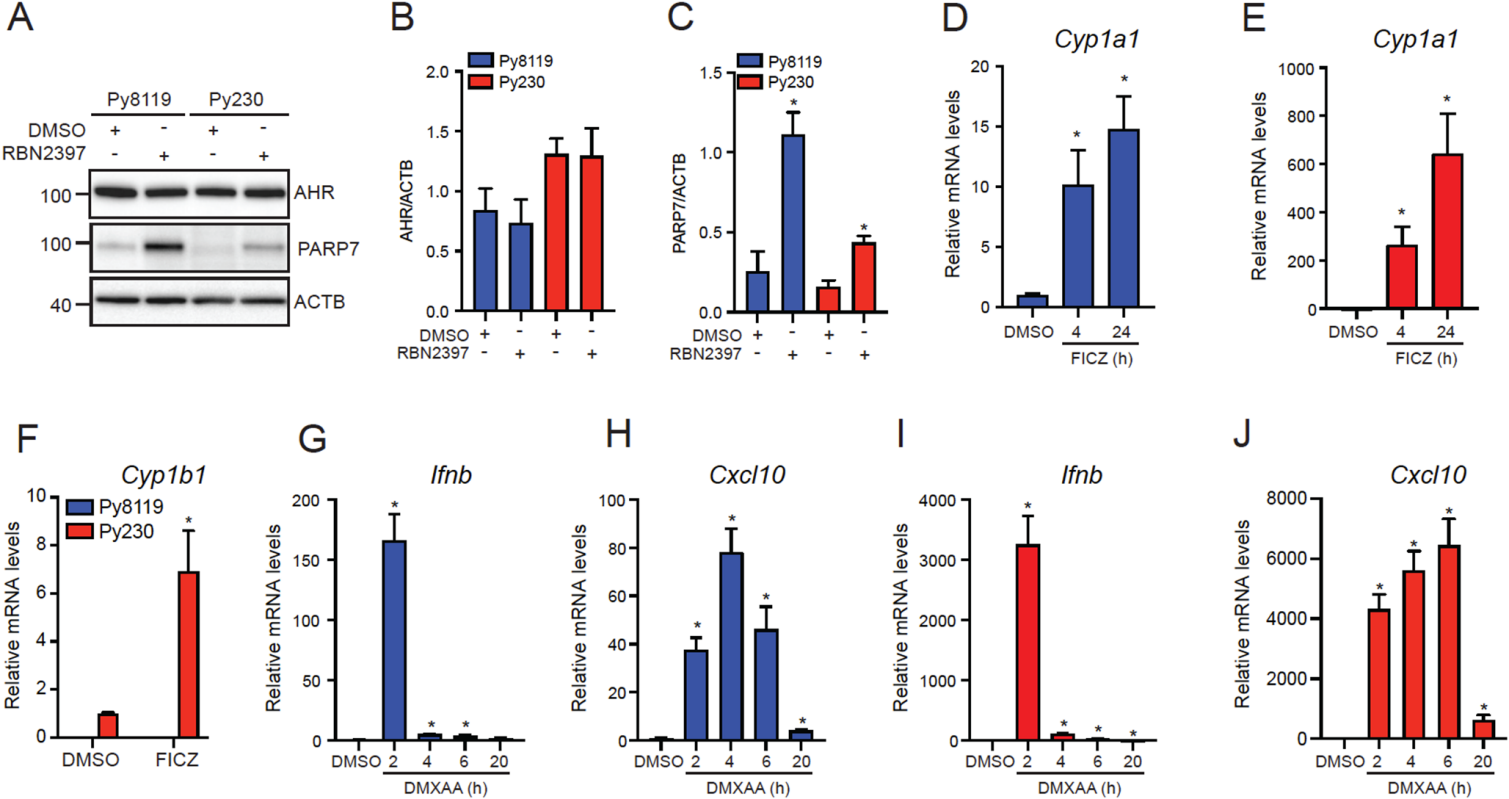
Py8119 and Py230 cells have functional AHR and IFN-I signalling pathways**.** (**A**) Py8119 and Py230 cells treated with DMSO or 100 nM RBN2397 for 24 h. Representative image of *n* = 3, 20 µg of protein were loaded. Quantification of western blots for AHR (**B**) and PARP7 (**C**). *Cyp1a1* mRNA levels in Py8119 (**D**) and Py230 (**E**) cells treated with DMSO or 10 nM FICZ for 4–24 h. (**F**) *Cyp1b1* mRNA levels in Py230 and Py8119 cells treated with DMSO or 10 nM FICZ for 4 h. Fold change normalized to Py230 treated with DMSO. (**G**) *Ifnb* and (**H**) *Cxcl10* mRNA levels in Py8119 treated with 10 µg/ml DMXAA for the times indicated. (**I**) *Ifnb* and (**J**) *Cxcl10* mRNA levels in Py230 treated with 10 µg/ml DMXAA over a time course. *p < 0.05 compared with DMSO determined by unpaired student’s *t*-test, *n* = 3

Here, we used Py8119 and Py230 cells which were isolated from spontaneous tumours in MMTV-PyMT C57BL/6 female mice [33]. Py8119 cells are mesenchymal-like and are classified a triple negative breast cancer line, while Py230 cells as luminal-like breast cancer type. Both cell lines are routinely used as breast cancer models, but their sensitivity to RBN2397, as well as the responsiveness of their AHR and STING-induced IFN-I signalling pathways have not been described. To this end, we first determined the AHR and PARP7 protein levels in both cell lines. Immunoblotting revealed that Py8119 and Py230 cells expressed similar AHR levels. Since inhibition of PARP7 catalytic activity stabilizes PARP7 protein levels [12], we treated cells with 100 nM RBN2397 to inhibit PARP7 activity allowing for efficient detection of PARP7 protein. Py8119 cells were found to express higher PARP7 levels compared with Py230 cells (Fig. 1A and C). We next examined the AHR responsiveness of both cell lines by determining *Cyp1a1* and *Cyp1b1* mRNA levels after treatment with 10 nM of the AHR agonist FICZ for 4 h and 24 h. FICZ increased *Cyp1a1* mRNA levels in both cell lines, but the increase was approximately 20-fold higher in Py230 cells at both time points (Fig. 1D and E). *Cyp1b1* mRNA levels were increased by FICZ in Py230 cells; however, no *Cyp1b1* was detected in Py8119 cells (Fig. 1F**)**.

To investigate STING-induced IFN-I signalling, we treated Py8119 and Py230 cells with DMXAA, an activator of murine, but not human STING, for 2 h to 20 h. In Py8119 cells, *Ifnb* mRNA levels reached a maximum increase after 2 h DMXAA treatment before decreasing after 4 h, while *Cxcl10* mRNA levels increased after 2 h and peaked at 4 h before decreasing at 6 h and further decreasing at 20 h (Fig. 1G and H). Similar findings were observed in DMXAA-treated Py230 cells, except that greater fold increases in *Ifnb* and *Cxcl10* levels were detected in Py230 cells (Fig. 1I and J).

### RBN2397 enhances AHR agonist-dependent signalling and stabilizes nuclear PARP7 protein levels

**Fig. 2:**
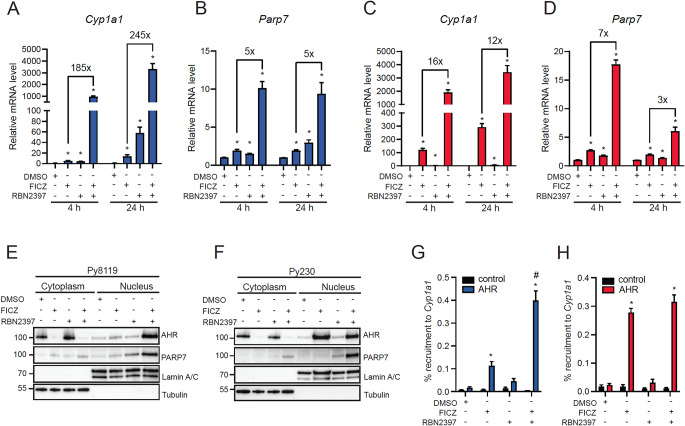
RBN2397 increases AHR signalling in combination with FICZ and causes nuclear trapping of PARP7. (**A**) *Cyp1a1* and (**B**) *Parp7* mRNA levels in Py8119 cells treated with DMSO, 10 nM FICZ, 100 nM RBN2397 or their combination, for 4–24 h. (**C**) *Cyp1a1* and (**D**) *Parp7* mRNA levels in Py230 cells treated with DMSO, 10 nM FICZ, 100 nM RBN2397 or combination, for 4–24 h. * *p* < 0.05 compared with DMSO, *n* = 3. Cytosolic and nuclear extracts of Py8119 (**E**) and Py230 (**F**) cells treated with DMSO, 10 nM FICZ, 100 nM RBN2397 or combination for 2 h. Representative image of *n* = 2, 10 µg of protein were loaded. Recruitment of AHR to *Cyp1a1* after 1 h treatment with FICZ, RBN2397 or their combination in (**G**) Py8119 and (**H**) Py230 cells. * *p* < 0.05 compared with AHR DMSO treatment. # *p* < 0.05 compared with AHR FICZ treatment, *n* = 3

We previously reported that PARP7 negatively regulates AHR and that RBN2397 enhances AHR agonist-dependent signalling [10, 12]. To examine how RBN2397 affects AHR signalling, Py8119 and Py230 cells were treated with FICZ, RBN2397 alone and in combination for 4 h and 24 h before determining *Cyp1a1* and *Parp7* levels. In Py8119 cells, FICZ resulted in a 5-fold and a 13-fold increase in *Cyp1a1* mRNA levels at 4 h and 24 h, respectively (Fig. 2A). Treatment with 100 nM RBN2397 caused a 4-fold increase at 4 h and a 57-fold increase at 24 h in *Cyp1a1* levels. To the best of our knowledge, RBN2397 is not an AHR agonist, thus the RBN2397-dependent increases in *Cyp1a1* might be due to endogenous or medium derived AHR ligand stimulation after inhibition of PARP7. Combination treatment with FICZ + RBN2397 resulted in synergistic increases in *Cyp1a1* levels at both time points. A similar expression pattern was observed for *Parp7* with FICZ causing a 2-fold increase at both 4 and 24 h, while RBN2397 resulted in a 1.5- and 3-fold at 4 h and 24 h, respectively (Fig. 2B). Combined FICZ and RBN2397 treatment increased *Parp7* levels 10-fold at both time points. In Py230 cells, FICZ alone strongly induced *Cyp1a1* levels 120- and 293-fold at 4 h and 24 h, respectively. RBN2397 resulted in a 2-fold increase at 4 h and a 7-fold increase at 24 h in *Cyp1a1* levels (Fig. 2C). Combined treatment with FICZ and RBN2397 synergistically increased *Cyp1a1* levels at both time points. For *Parp7*, FICZ caused an approximate 2-fold increased at both 4 and 24 h, while RBN2397 resulted in a 1.7- and 1.4-fold at 4 h and 24 h, respectively (Fig. 2D). It is worth noting that in Py8119 cells FICZ + RBN2397 increased *Cyp1a1* mRNA levels 185- and 245-fold higher than FICZ treatment alone at 4 h and 24 h, respectively. However, for Py230 cells FICZ + RBN2397 cotreatment caused a 16- and 12-fold higher increase in *Cyp1a1* levels at 4 h and 24 h, respectively, compared with FICZ treatment.

Since ligand induced nuclear translocation is an important and necessary component of AHR signalling [35], we examined how FICZ and RBN2397 affected the cytosolic-nuclear localization of AHR and PARP7. Cytosolic AHR levels were decreased after treatment with FICZ and FICZ + RBN2397 in Py8119 cells (Fig. 2E, Supplementary Figure S1). A small increase in nuclear AHR was observed after FICZ treatment, suggesting that the reduced AHR protein levels were due to agonist-induced proteolytic degradation. FICZ + RBN2397 treatment resulted in a marked increase in nuclear AHR, which was consistent with the increased AHR-regulated gene expression shown in Fig. 2A and B. RBN2397, FICZ and FICZ + RBN2397 had minimal impact on cytosolic PARP7 levels. Nuclear PARP7 levels were slightly increased by FICZ, more strongly increased with RBN2397, while the combined treatment caused a clear increase in PARP7 levels. For Py230 cells, cytosolic AHR levels were decreased with a concomitant increase in nuclear AHR levels by nuclear translocation after treatment with FICZ and FICZ + RBN2397 (Fig. 2F, Supplementary Figure S2). This was consistent with the increased AHR-regulated gene expression in Py230 cells shown in Fig. 2C and D. Similarly to Py8119 cells, the treatments except for FICZ + RBN2397 had minimal impact on cytosolic PARP7 levels in Py230 cells. Nuclear PARP7 levels were not affected by FICZ and increased slightly by RBN2397, while the combined treatment caused an increase in PARP7 levels. To determine the ability of FICZ and RBN2397 to induce recruitment of AHR to *Cyp1a1* chromatin immunoprecipitation (ChIP) assays were done after treating cells with ligands for 1 h. The 1 h time point was chosen because we have previously reported that AHR recruitment peaks between 45 and 90 min [36]. In Py8119 cells, ChIP assays showed that FICZ induced a weak but statistically significant increase in AHR recruitment to *Cyp1a1*, which was enhanced with RBN2397 cotreatment (Fig. 2G). FICZ strongly induced AHR binding to *Cyp1a1* in Py230 cells, but this was unaffected by RBN2397 cotreatment at the time point examined (Fig. 2H). Taken together, these data confirm the negative regulation of AHR by PARP7, and that PARP7 has a more prominent inhibitory effect on AHR signalling in Py8119 compared with Py230 cells. Our findings also show that RBN2397 stabilizes nuclear PARP7 suggesting that inactive PARP7 becomes trapped in the nuclear compartment.

### RBN2397 differentially affects DMXAA-induced IFN-I signalling in Py8119 and Py230 cells

**Fig. 3:**
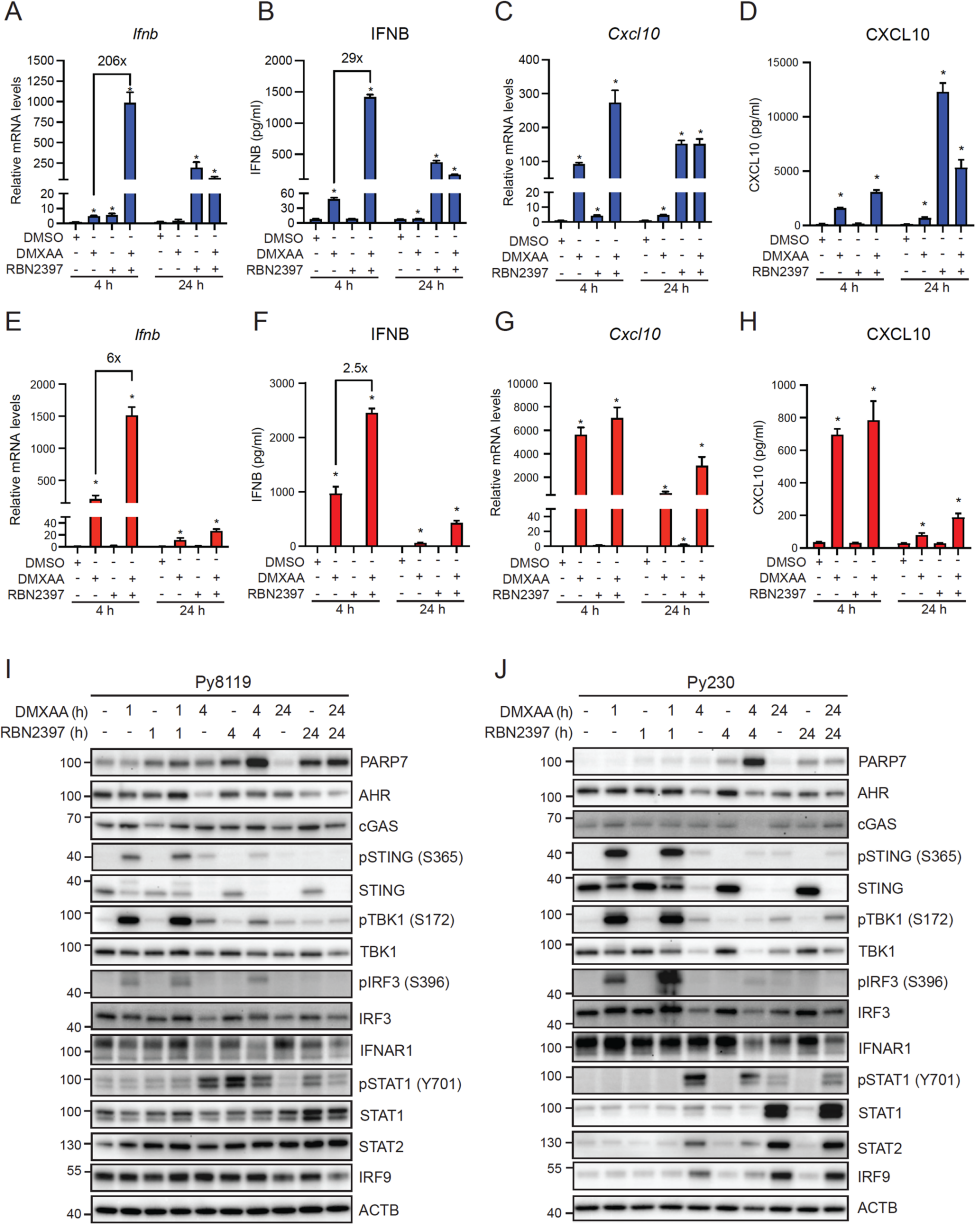
RBN2397 causes a longer and more sustained increase in IFN-I signalling in Py8119 cells than in Py230 cells. (**A**) *Ifnb* mRNA levels and (**B**) IFNB protein concentration from Py8119 cells. (**C**) *Cxcl10* mRNA level and (**D**) protein concentration from Py8119 cells. (**E**) *Ifnb* mRNA and (**F**) protein in Py230 cells. (**G**) *Cxcl10* mRNA and (**H**) protein in Py230 cells. Py8119 and Py230 cells were treated 4–24 h with DMSO, 10 µg/mL DMXAA, 100 nM RBN2397 or combination. *p < 0.05 compared with DMSO tested by student’s *t*-test for each time point, *n* = 3. Western blot of IFN-I signalling cascade in Py8119 (**I**) and Py230 (**J**) cells treated with DMSO (24 h), 10 µg/mL DMXAA, 100 nM RBN2397 or combination for 1 h, 4–24 h. Representative image of *n* = 2, 15 µg of protein were loaded

To characterize IFN-I signalling in both cell lines we treated cells with 10 µg/mL DMXAA, 100 nM RBN2397 or in combination for 4–24 h. After 4 h treatment, DMXAA caused 4.8-fold and RBN2397 caused 5.6-fold increases in *Ifnb* mRNA levels in Py8119 cells (Fig. 3A). DMXAA + RBN2397 induced a synergistic increase in *Ifnb* levels that was more than 200-fold that induced by DMXAA alone. DMXAA failed to increase *Ifnb* levels at 24 h. RBN2397 caused a robust 197-fold increase in *Ifnb* levels at 24 h, which was reduced by DMXAA cotreatment. The changes in *Ifnb* mRNA levels were reflected in ELISA studies except that the synergistic increase in IFNB levels at 4 h was only 29-fold higher compared with DMXAA alone (Fig. 3B). In line with the *Ifnb* levels, DMXAA induced *Cxcl10* mRNA levels at 4 h which were increased (∼3-fold) with RBN2397 cotreatment (Fig. 3C). RBN2397 strongly increased *Cxcl10* mRNA levels at 24 h and this was unaffected by cotreatment with DMXAA. CXCL10 protein levels mirrored *Cxcl10* mRNA levels at 4 h, but the impact of RBN2397 alone on CXCL10 levels was more evident at 24 h (Fig. 3D). Since AHR has been reported to directly regulate IFN-I signalling, we determined the ability of FICZ and BAY2416964, an AHR antagonist, to affect *Ifnb* or *Cxcl10* expression levels (Supplementary Figure S3). Treatment with 10 nM FICZ or 1 µM BAY2416964 for 4–24 h did not affect *Ifnb* mRNA levels. *Cxcl10* levels were also unaffected by either treatment at 4 h; however, at 24 h FICZ caused a slight decrease while BAY2416964 caused a weak increase in *Cxcl10* mRNA levels.

For Py230 cells, DMXAA increased *Ifnb* mRNA levels at 4 h which were further increased with RBN2397 cotreatment. RBN2397 alone failed to induce *Ifnb* levels at 4 h. *Ifnb* levels were not increased with DMXAA and RBN2397 + DMXAA treatment for 24 h (Fig. 3E). IFNB levels increased after 4 h treatment with DMXAA, which were increased 2.5-fold with RBN2397 cotreatment (Fig. 3F). DMXAA induced *Cxcl10* mRNA levels in Py230 cells at 4 h, but they were unaffected by the addition of RBN2397 (Fig. 3G). After 24 h incubation, the DMXAA-induced *Cxcl10* levels were further increased with RBN2397 cotreatment. CXCL10 protein levels reflected the observed changes in *Cxcl10* mRNA at both time points (Fig. 3H). Treatment with FICZ or BAY2416964 did not affect *Ifnb* levels at 4 h, while FICZ caused a less than 2-fold increase in *Ifnb* expression at 24 h. Cxcl10 levels were slightly increased by FICZ and BAY at 4 h, but neither compound affected Cxcl10 levels at 24 h (Supplementary Figure S3). Collectively, these findings show that similar for AHR signalling pathway, PARP7 has a more prominent inhibitory effect on IFN-I signalling in Py8119 compared with Py230 cells.

We then investigated the effect of DMXAA and RBN2397 on the expression levels and phosphorylation status of proteins in the IFN-I signalling cascade. In Py8119 cells (Fig. 3I, Supplementary Figure S4), PARP7 protein increased upon treatment with RBN2397 for all time points, with the highest signal induced by DMXAA + RBN2397 at 4 h. AHR levels decreased at 4 h with DMXAA, and at 24 h with DMXAA or DMXAA + RBN2397. No differences in cGAS levels were observed at any time point. DMXAA induced STING phosphorylation (pSTING, S365) peaked at 1 h and was independent of RBN2397. In agreement with a previous study, native STING levels were inversely correlated with pSTING levels [37]. The phosphorylation patterns of TBK1 (S172) and IRF3 (S396) closely followed those of pSTING, while native TBK1 and IRF3 expression levels were relatively constant throughout the time and treatment course. IFNAR1 levels were also relatively constant throughout the time and treatment course. pSTAT1 (Y701) was detected in all 4 h treatments and at 24 h treatment with RBN2397. STAT1 levels were constant throughout all time points with elevated levels at 24 h treatments with RBN2397 and DMXAA + RBN2397. STAT2 and IRF9 levels were stable at all time points and unaffected by treatment, except for reduced IRF9 levels at 24 h DMXAA or DMXAA + RBN2397 treatments. For Py230 cells (Fig. 3J, Supplementary Figure S5), we observed similar expression patterns for PARP7 and AHR proteins as was observed for Py8119 cells; although, the levels of PARP7 were lower in Py230 cells. cGAS levels were relatively constant but lower compared with Py8119 cells. pSTING and STING expression patterns were equivalent those of Py8119 cells, but higher levels were observed in Py230 cells. pTBK1 and TBK1 levels were comparable to those seen in Py8119 cells but with notably lower levels induced by DMXAA at 4 h. DMXAA induced pIRF3 levels at 1 h which were increased with RBN2397 cotreatment. Native IRF3 levels were similar to those of Py8119 cells. IFNAR1 levels were relatively constant throughout the time and treatment course. pSTAT1 was increased at 4 h and 24 h with DMXAA and DMXAA + RBN2397. No pSTAT1 was detected after RBN2397 treatment. Compared with Py8119 cells, native STAT1, STAT2 and IRF9 proteins were generally lower in Py230 cells with clear increases at 24 h with DMXAA alone and with RBN2397 cotreatment. Collectively these data show that Py8119 cells are more responsive to RBN2397-dependent increases in IFN-I signalling, while DMXAA induces a greater STING-induced IFN-I response in Py230 cells. Combination treatment of RBN2397 + DMXAA also resulted in greater increases in *Ifnb* and *Cxcl10* levels compared to DMXAA alone in Py8119 than in Py230 cells.

### Py8119 but not Py230, 4T1 or EO771 cells are sensitive to the antiproliferative effect of RBN2397

**Fig. 4:**
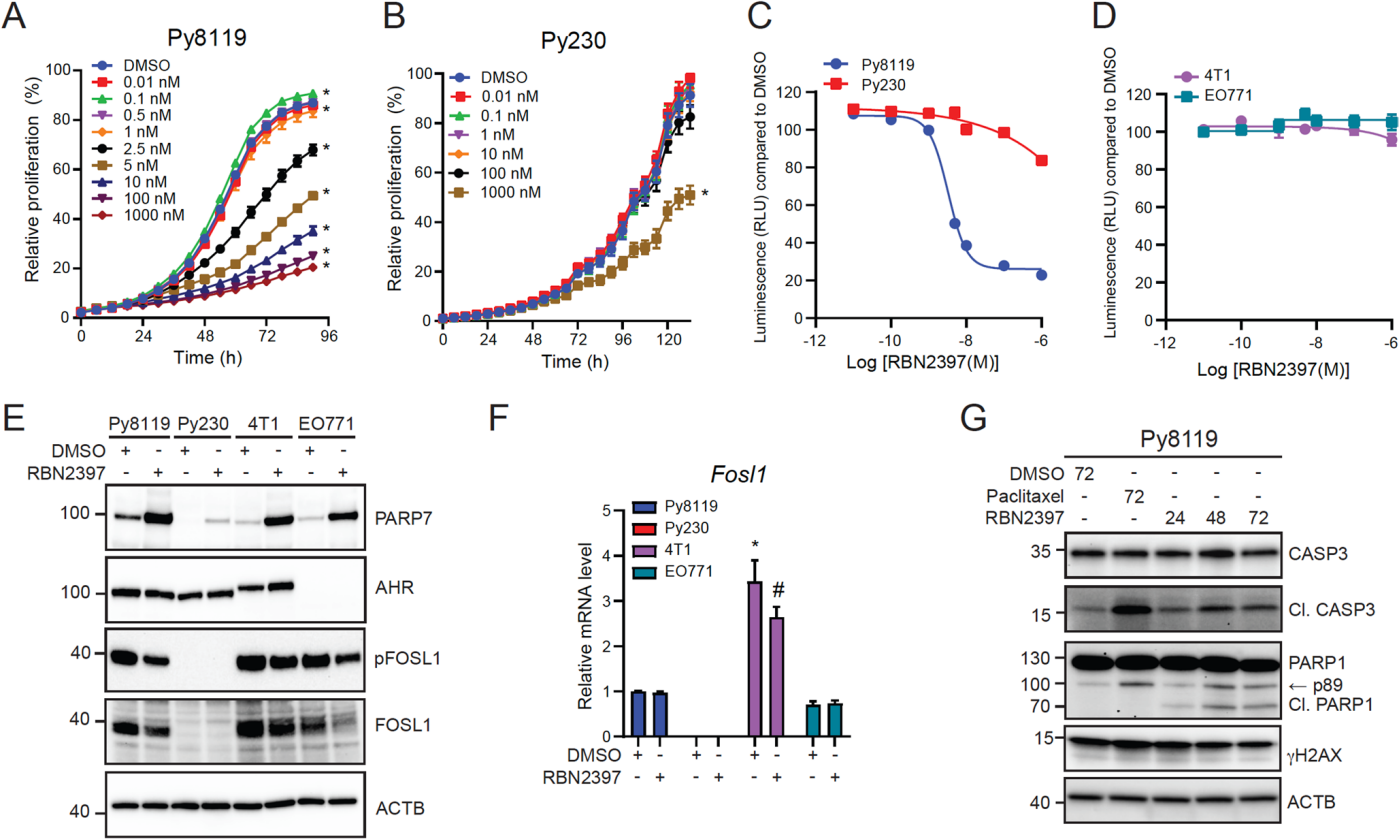
Py8119 but not Py230, 4T1 or EO771 cells are sensitive to the antiproliferative effect of RBN2397. (**A**) Py8119 and (**B**) Py230 cells treated with different concentrations of RBN2397 over 90–132 h, respectively, measured by IncuCyte. *Significance from DMSO, *n* = 8. (**C**) IC_50_ curve of Py8119 and Py230 cells treated with 0.01 to 1000 nM of RBN2397 normalized to DMSO = 100, *n* = 5. (**D**) 4T1 and EO771 cells treated with increasing concentrations of RBN2397, *n* = 8. (**E**) Py8119, Py230, 4T1 and EO771 cells treated 24 h with DMSO or 100 nM RBN2397. Representative image of *n* = 2, 30 µg protein loaded. (**F**) *Fosl1* mRNA level in Py8119, Py230, 4T1 and EO771 cells treated 24 h with DMSO or 100 nM RBN2397, normalized to Py8119 DMSO. * *p* < 0.05 compared with Py8119 DMSO, # *p* < 0.05 compared with cell line matched DMSO, *n* = 3. (**G**) Py8119 treated with DMSO, 100 nM paclitaxel or 100 nM RBN2397 for 24, 48 and 72 h reveal cleavage (Cl.) of CASP3 and PARP1 as a sign of apoptosis and gH2AX as a marker of DNA damage. Representative image of *n* = 3, 15 µg of protein were loaded

To determine whether Py8119 or Py230 cells are sensitive to the antiproliferative effects of PARP7 inhibition, cells were treated with increasing concentrations of RBN2397 and their proliferation monitored. Py8119 cells were more sensitive to the antiproliferative effects of RBN2397 than Py230 cells when determined by IncuCyte and by CellTiter Glo™ assays (Fig. 4A and B). The calculated absolute IC_50_ value for RBN2397 that caused 50% inhibition of Py8119 cell proliferation was 5.6 ± 0.52 nM (Fig. 4C, Supplementary Table S1). Reduced proliferation of Py230 cells was only observed at 1 µM RBN2397 (Fig. 4B and C). We next determined how RBN2397 affected the proliferation of 4T1 and EO771 cells, two mouse mammary cancer cell lines that are commonly used in preclinical cancer studies. However, both 4T1 and EO771 cells were insensitive to RBN2397 (Fig. 4D). Immunoblotting revealed that 4T1 and EO771 cells had similar levels of PARP7 compared with Py8119 cells, while 4T1 but not EO771 cells expressed AHR (Fig. 4E, Supplementary Figure S6). 4T1 cells are derived from BALB/c mice which express *Ahr*^*b−2*^ allele (104 kDa) which is consistent with the higher molecular weight form of AHR in 4T1 compared with in Py8119 and Py230 cells that express *Ahr*^*b−1*^ allele (95 kDa). Since the antiproliferative effect of RBN2397 is also reported to be through IRF3-dependent apoptosis resulting from reduced FOSL1 levels but no change in pFOSL1 [18], we determined their levels in the four cell lines. Native FOSL1 and pFOSL1 levels were reduced with RBN2397 sensitive Py8119 cells, and no FOSL1 or pFOSL1 was detected in RBN2397 resistant Py230 cells. Despite their insensitivity to RBN2397, 4T1 and EO771 expressed similar FOSL1 and pFOSL1 levels compared with Py8119 cells, and the levels of both were reduced by RBN2397. *Fosl1* mRNA levels were 11- and 2-fold higher in 4T1 and EO771, respectively, compared with Py8119 cells, whereas *Fosl1* levels were ~ 100-fold lower in Py230 cells. *Fosl1* levels were unaffected by RBN2397 treatment of Py8119 and EO771 cells but slightly reduced in 4T1 cells (Fig. 4F). We then determined if the PARP7-dependent reduction in the proliferation of Py8119 occurred through apoptosis. The chemotherapeutic and apoptosis inducer, paclitaxel, was used as a positive control. Paclitaxel and RBN2397 induced cleavage of caspase-3 (CASP3) and the 89 kDa peptide fragment of cleaved PARP1 were observed in Py8119 cells, suggesting the antiproliferative effect of RBN2397 is due to increased apoptosis (Fig. 4G, Supplementary Figure S7). A lower molecular weight fragment of PARP1 of ∼70 kDa was also detected in RBN2397 but not paclitaxel treated samples. Native CASP3 and PARP1 levels were unaffected by treatment. To determine if any of the ligand treatments affected DNA damage, we analysed gH2AX levels. The levels of gH2AX were relatively high in Py8119 cells but unaffected by paclitaxel or RBN2397 treatments.

### DMXAA sensitizes Py230 cells to the antiproliferative effects of RBN2397 by inducing autophagy

**Fig. 5:**
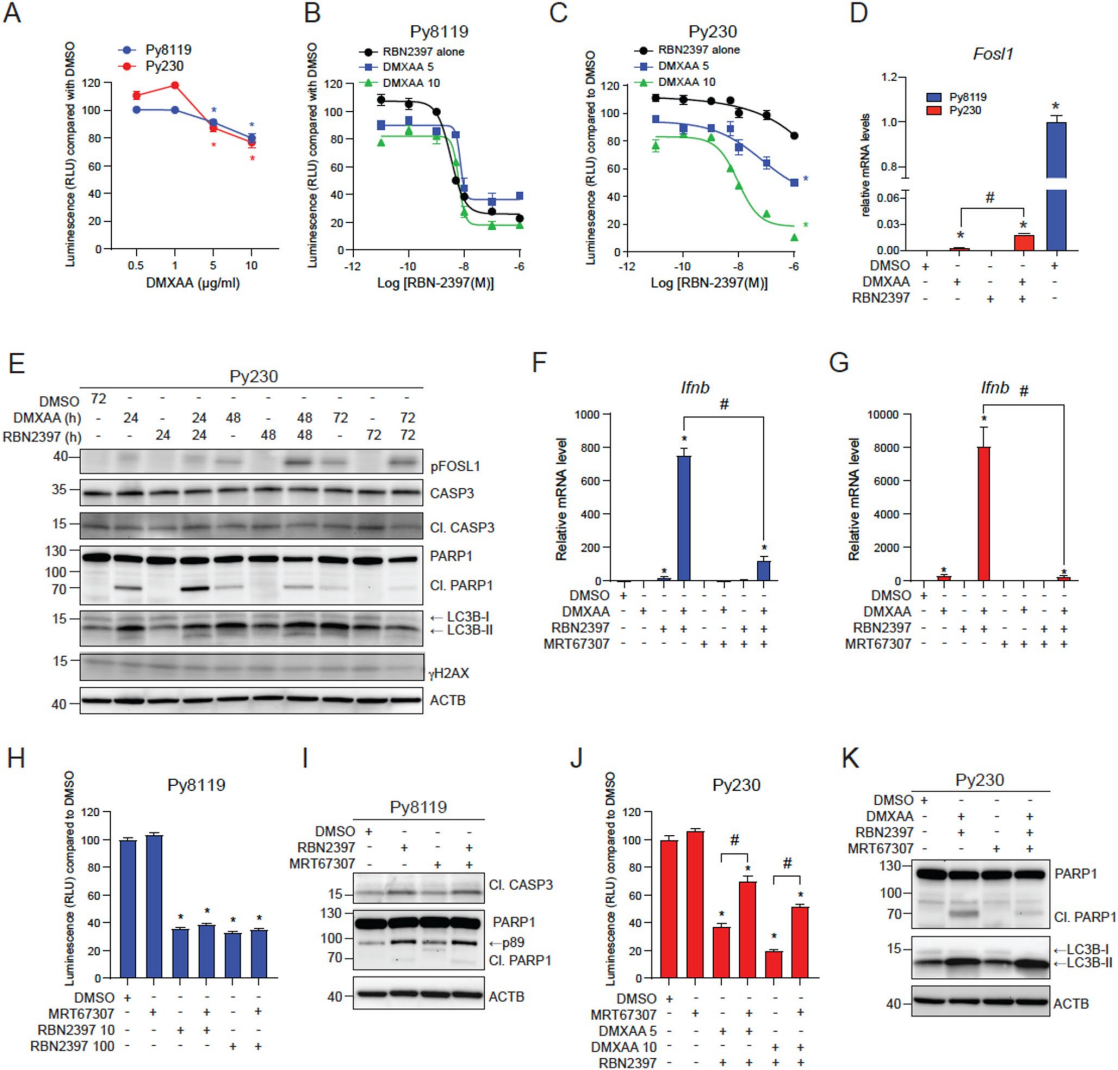
The antiproliferative effect of RBN2397 is dependent on DMXAA-induced IFN-I signalling in Py230 cells. (**A**) Proliferation of Py8119 and Py230 cells treated with 0.5, 1, 5, and 10 µg/ml DMXAA. * Significant decrease compared to DMSO, blue for Py8119, red for Py230 cells. IC_50_ of Py8119 (**B**) or Py230 (**C**) cells treated with 5–10 µg/mL DMXAA in combination with 0.01 to 1000 nM RBN2397. RBN2397 treatment alone from Fig. 4C is added for comparison. *Significant decrease compared RBN2397 tested by area under curve, *n* = 8. (**D**) *Fosl1* mRNA levels in Py230 cells treated with DMSO, 10 µg/mL DMXAA, 100 nM RBN2397 or combination for 48 h, and DMSO treated Py8119. (**E**) Py230 cells treated with DMSO, 10 µg/mL DMXAA, 100 nM RBN2397 or combination for 24 h, 48–72 h. Representative image of *n* = 3, 30 µg protein loaded. *Ifnb* mRNA level in Py8119 (**F**) and Py230 (**G**) cells treated with 10 µg/mL DMXAA, 100 nM RBN2397, 1 µM MRT67307 or combinations for 4 h. (**H**) Proliferation of Py8119 cells treated with DMSO, 1 µM MRT67307, 10 nM RBN2397, 100 nM RBN2397 or combination, *n* = 4. (**I**) Western blot of Py8119 cells treated 48 h with DMSO, 1 µM MRT67307, 100 nM RBN2397, or combination. Representative image of *n* = 2, 30 µg of protein was loaded. (**J**) Proliferation of Py230 cells treated with DMSO, 5–10 µg/mL DMXAA in combination with 100 nM RBN2397, 1 µM MRT67307, or combinations of DMXAA, RBN2397 and MRT67307, *n* = 2. (**K**) Western blot of Py230 cells treated 24 h with DMSO, 10 µg/mL DMXAA + 100 nM RBN2397, 1 µM MRT67307, or combinations. Representative image of *n* = 2, 30 µg of protein were loaded. *p < 0.05 compared with DMSO. #*p* < 0.05 compared with the indicated treatments, *n* = 4

We next exposed Py8119 and Py230 cells to increasing concentrations of DMXAA and RBN2397 and monitored their proliferation. Slight, but significant, dose dependent decreases in Py8119 and Py230 cell proliferation were observed after treatment with 5–10 µg/mL DMXAA (Fig. 5A; Supplementary Figure S4). No significant differences in the IC_50_ values between Py8119 cells treated with both 5 and 10 µg/mL DMXAA in combination with RBN2397 compared with RBN2397 alone (Fig. 5B; Supplementary Table S1). On the other hand, DMXAA sensitized Py230 cells to RBN2397 with an IC_50_ value of 9.7 ± 2.2 nM for combined RBN2397 + 10 µg/mL DMXAA (Fig. 5C; Supplementary TableS2). We next determined if DMXAA increased *Fosl1* levels in Py230 cells which could contribute to their sensitive to RBN2397 in the presence of STING activation. DMXAA treatment for 48 h increased *Fosl1* levels which were further increased with RBN2397 co-treatment, but the relative levels were over 500-fold lower compared with DMSO treated Py8119 cells (Fig. 5D). RBN2397 alone did not affect *Fosl1* levels. We were unable to detect any increases in native FOSL1 (data not shown). This was most likely due to the low FOSL1 levels and limitations of the anti-FOSL1 antibody used. However, DMXAA resulted in a time-dependent increase in pFOSL1, which was increased with RBN2397 co-treatment. RBN2397 alone did not affect pFOSL1 levels. Despite the increased pFOSL1 and decreased proliferation, no increases in cleaved CASP3 or the p89 fragment of PARP1 were detected with any treatment in Py230 cells, suggesting the decreased proliferation was not due to apoptosis (Fig. 5E, Supplementary Figure S9). As observed in RBN2397-treated Py8119 cells, we detected the PARP1 fragment around 70 kDa after 24 h treatment of DMXAA which increased with DMXAA + RBN2397 before decreasing after 48 h and further decreased at 72 h. Since IFN-I responses also induce autophagy, we determined the relative levels of LC3B-I and LC3B-II [38]. Increased levels of LC3B-II were observed in Py230 cells treated with DMXAA alone at all time points, and for DMXAA + RBN2397 cotreatment at 24 h and 48 h. The levels of gH2AX levels were also determined. The gH2AX levels were lower compared with Py8119 cells gH2AX levels, but unaffected by paclitaxel or RBN2397. Collectively, these findings suggest that the reduced cell growth of Py230 cells may be due to increased autophagy.

To determine if the antiproliferative effect of PARP7 inhibition in Py8119 and DMXAA + RBN2397 in Py230 cells were dependent on TBK1-activated IFN signalling cells were treated with the TBK1 inhibitor, MRT67307. As expected, MRT67307 reduced DMXAA + RBN2397 dependent increases in *Ifnb* mRNA levels in Py8119 and Py230 cells (Fig. 5F and G). MRT67307, however, did not prevent the antiproliferative effect of RBN2397 in Py8119 cells (Fig. 5H), and the protein levels of CASP3 and PARP1 cleavage fragments were also unaffected by TBK1 inhibition (Fig. 5I, Supplementary Figure S10). MRT67307 treatment partially reduced the anti-proliferative effects of DMXAA + RBN2397 treatment in Py230 cells (Fig. 5J). Interestingly, the ∼70 kDa fragment of PARP1 decreased with TBK1 inhibition, while LC3B-II levels were unchanged (Fig. 5K, Supplementary Figure S11). These findings suggest that the antiproliferative and apoptotic effects of RBN2397 in Py8119 cells are independent of STING-induced TBK1 activation. However, STING activation sensitized Py230 cells to the antiproliferative effects of RBN2397 through increased and perhaps dysregulated autophagy.

### Pharmacologic activation or Inhibition of AHR marginally affects RBN2397 sensitivity of Py8119 and Py230 cells

**Fig. 6:**
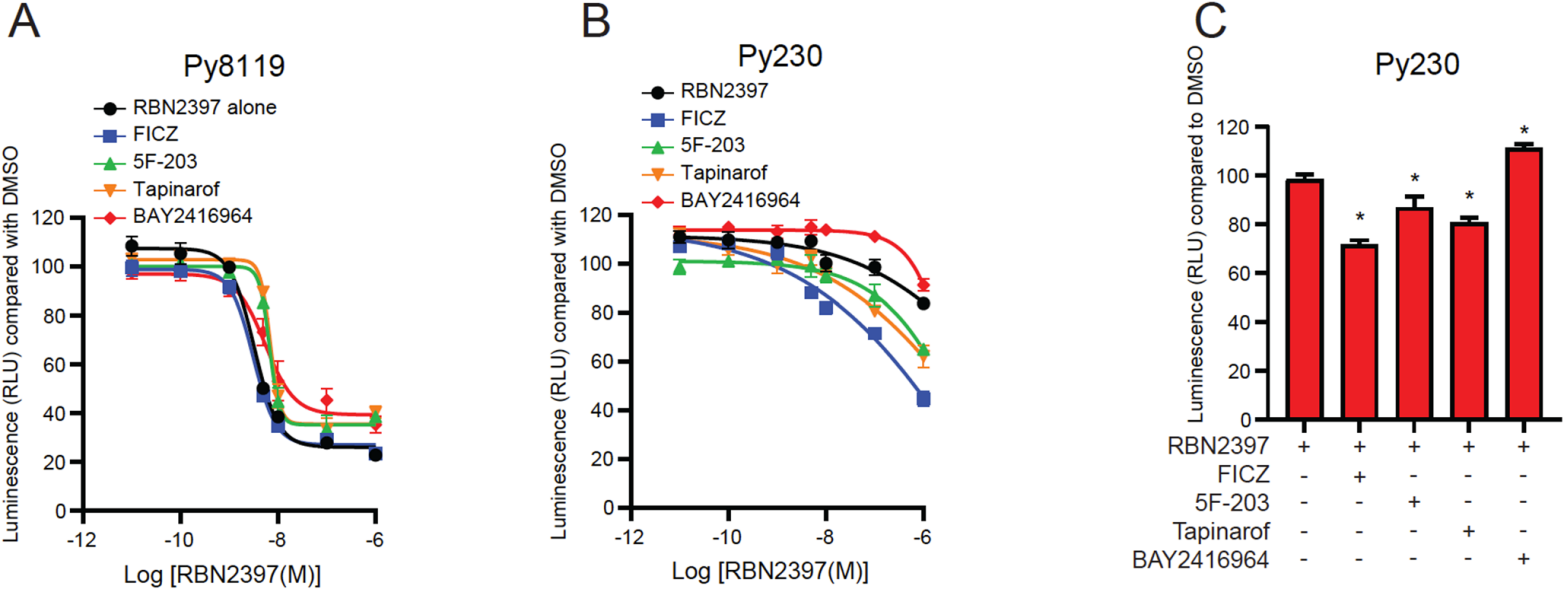
Activation of AHR slightly affect RBN2397 sensitivity. IC_50_ of Py8119 (**A**) or Py230 (**B**) cells treated with 10 nM FICZ, 1 µM 5F-203, 1 µM tapinarof, or 1 µM BAY2416964 in combination with 0.01 to 1000 nM RBN2397, *n* = 8. RBN2397 treatment alone from Fig. 4C is added for comparison. (**C**) Bar graph from Fig. 6B of Py230 cells treated with 100 nM RBN2397 alone or in combination with FICZ, 5 F-203, tapinarof, or BAY2416964. *p < 0.05 compared with RBN2397

Because AHR expression has been reported to be required for sensitivity to the antiproliferative effects of PARP7 inhibition, we next examined if activation or inhibition of AHR in combination with RBN2397 would affect the proliferation of Py8119 and Py230 cells. Cells were treated with fixed concentrations of the AHR agonists FICZ, 5 F-203 and tapinarof, or the AHR antagonist BAY2416964, in combination with increasing doses of RBN2397. 5 F-203 is reported to induce DNA damage and decrease growth of breast cancer cells [39], while tapinarof is an FDA approved AHR agonist used to treat psoriasis and atopic dermatitis and has been shown to increase the sensitivity of cancer cell lines to RBN2397 [30]. BAY2416964 is an AHR antagonist reported to reduce cancer cell growth and is currently in clinical trials (NCT04999202) [34, 40]. The IC_50_ values determined for combination treatment of FICZ, 5 F-203 or tapinarof with RBN2397 were not significantly different compared with the IC_50_ value for RBN2397 in Py8119 cells. A slight but significant increase in IC_50_ value for RBN2397 treated in combination with BAY2416964 was observed (Fig. 6A; Supplementary Table S1). For Py230 cells, none of the combined AHR agonists/antagonist and RBN2397 treatments were effective enough to allow for the calculation of an IC_50_ value (Fig. 6B). Nonetheless, compared to100 nM RBN2397, all AHR agonists decreased, while BAY2416964 slightly increased Py230 cell proliferation compared to 100 nM RBN2397 alone (Fig. 6C). Treatment with 10 nM RBN2397 in combination with the different ligands revealed a decrease only in combination with FICZ, while 1 µM RBN2397 resulted in similar findings to those for 100 nM RBN2397 (Supplementary Figure S12).

### *Ahr* loss increases STING-induced IFN-I signalling in Py8119 cells

**Fig. 7:**
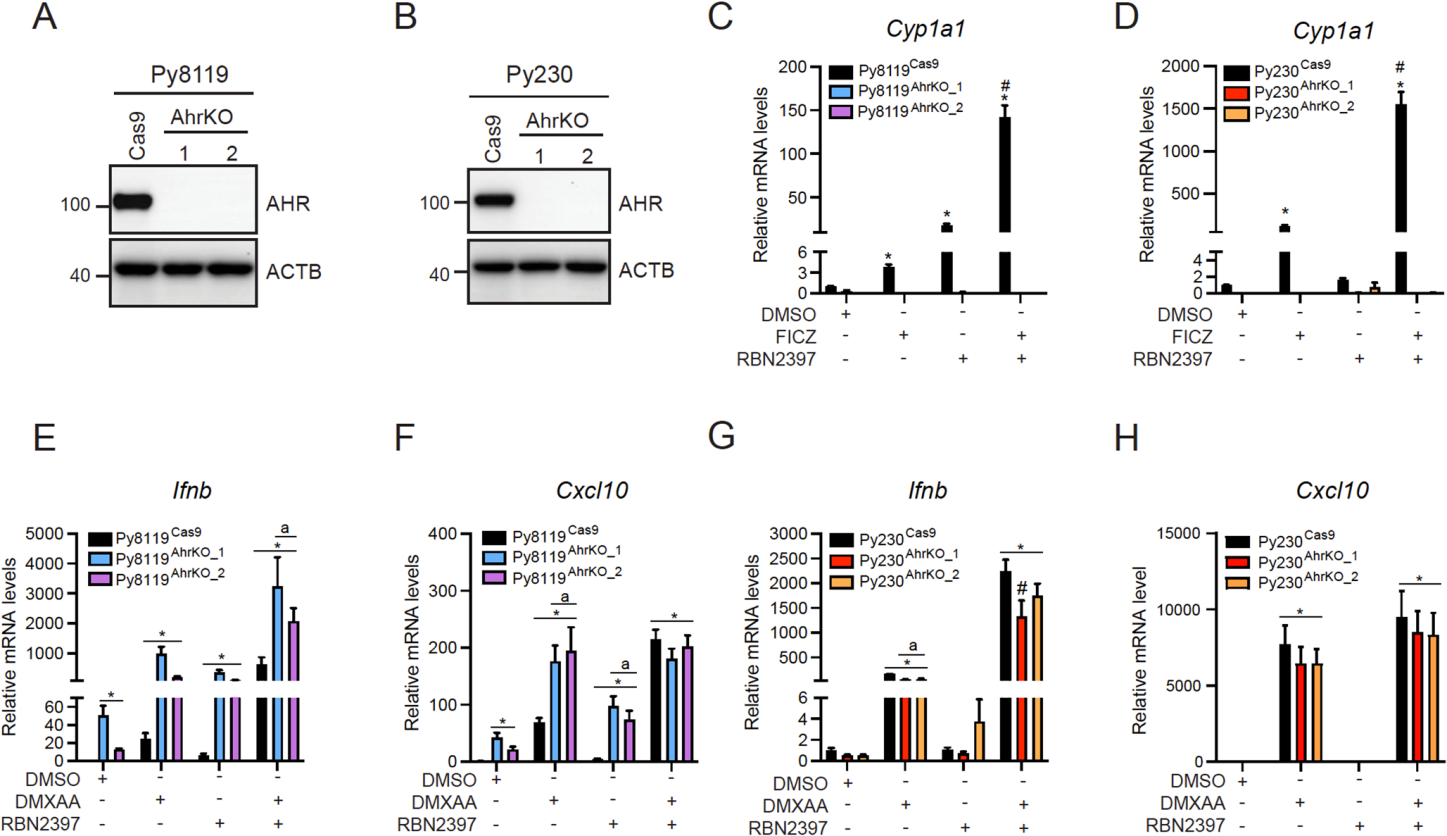
Loss of *Ahr* increases STING-induced IFN-I signalling in Py8119 cells, but not in Py230 cells. (**A**) Western blot of Py8119^Cas9^, Py8119^AhrKO_1^ and Py8119^AhrKO_2^ confirming loss of AHR in the AhrKO clones. (**B**) Western blot of Py230^Cas9^, Py230^AhrKO_1^ and Py230^AhrKO_2^ confirming loss of AHR in the AhrKO clones. 20 µg of protein were loaded. Representative images of *n* = 3. *Cyp1a1* mRNA levels in Py8119 (**C**) and Py230 (**D**) Cas9 and AhrKO clones. *p < 0.05 significantly increased from Cas9 treated with DMSO unpaired student’s *t*-test. Cells were treated 4 h with 10 nM FICZ, 100 nM RBN2397 or combination. (**E**) *Ifnb* and (**F**) *Cxcl10* mRNA levels in Py8119 and Py230 (**G**, **H**) Cas9 and AhrKO clones. Cells were treated with 10 µg/mL DMXAA, 100 nM RBN2397 or combination for 4 h. *p < 0.05 significantly increased from Cas9 treated with DMSO. #*p* < 0.05 compared with FICZ treated cells. ^a^*p* < 0.05 compared with Cas9 cells within the same treatment condition determined by one-way ANOVA. *n* = 3

To determine if loss of AHR affected STING-induced IFN-I signalling in the two cell lines, we generated Py8119 and Py230 AhrKO cell clones using CRISPR/Cas9. After clonal selection and DNA sequencing, we identified two independent AhrKO clones for Py8119 and Py230 cells in which frameshift mutations and premature stop codons were verified by DNA sequencing (Supplementary Table S13). These clones will be referred to as Py8119^AhrKO_1^, Py8119^AhrKO_2^, and Py230^AhrKO_1^, Py230^AhrKO_2^, with the empty vector control cells as Py8119^Cas9^ and Py230^Cas9^. Immunoblotting confirmed the lack of AHR protein in Py8119 and Py230 AhrKO cell lines (Fig. 7A and B). Treatment with FICZ alone increased *Cyp1a1* mRNA levels, which were synergistically increased in combination with RBN2397 in the Py8119 and Py230 Cas9 cells but not in their respective AhrKO clones (Fig. 7C and D). Constitutive *Ifnb* mRNA levels were increased in Py8119^AhrKO_1^ and Py8119^AhrKO_2^ compared with Py8119^Cas9^ cells. DMXAA, RBN2397 and their cotreatment caused a greater increase in *Ifnb* levels in both AhrKO clones compared with Py8119^Cas9^ cells (Fig. 7E). *Cxcl10* mRNA levels were increased in DMXAA- or RBN2397-treated Py8119^AhrKO_1^ and Py8119^AhrKO_2^ cells compared with similarly treated Py8119^Cas9^ cells. Cotreatment did not further increase *Cxcl10* levels in AhrKO clones compared with Py8119^Cas9^ cells (Fig. 7F). For Py230 cells, loss of AHR only had a minor effect on DMXAA and RBN2397 induced *Ifnb* and *Cxcl10* mRNA levels, with significant decreases observed for *Ifnb* levels in Py230^AhrKO_1^ with co-treatment (Fig. 7G and H).

**Fig. 8:**
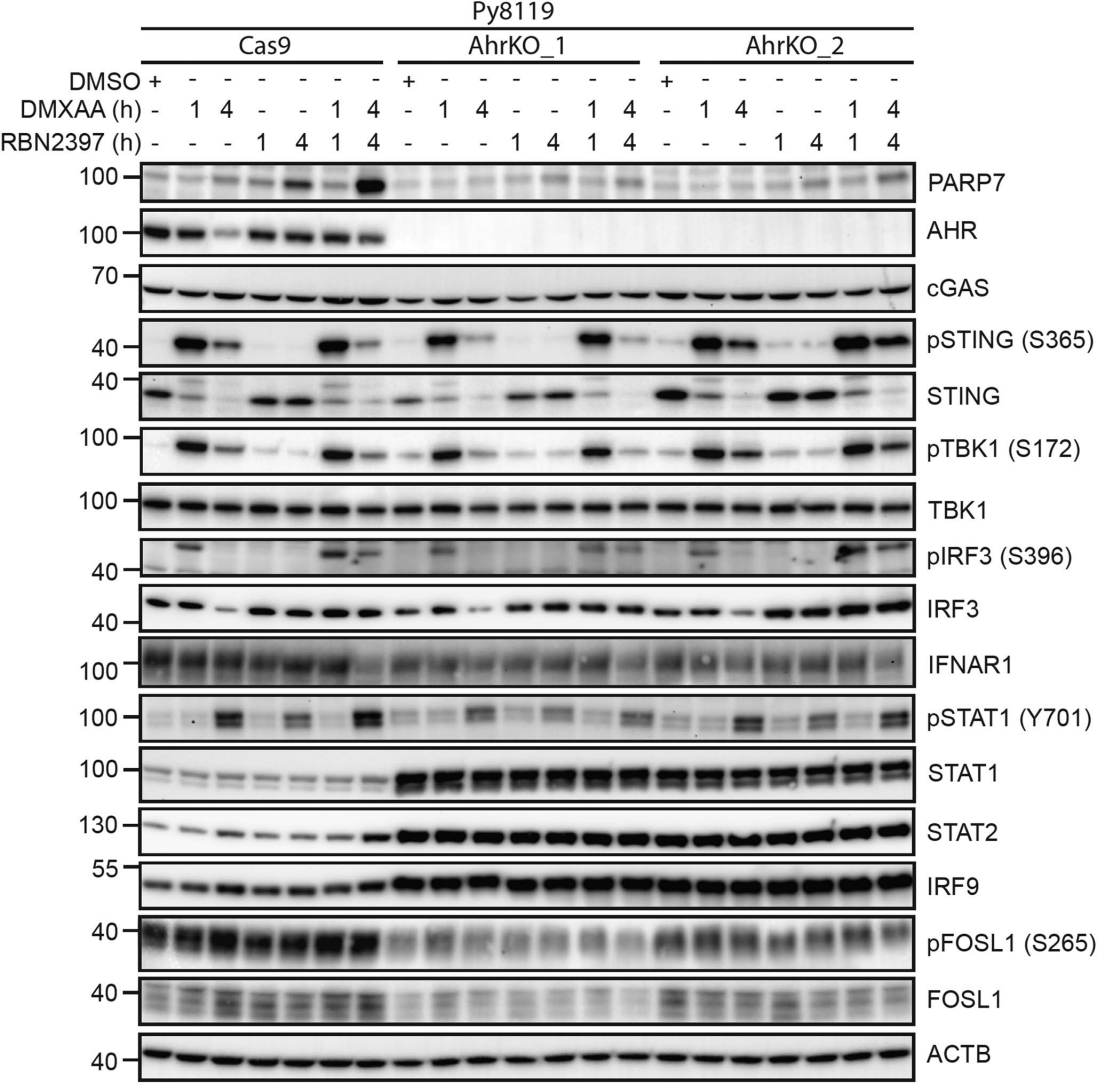
Loss of AHR in Py8119 cells increases unphosphorylated protein levels of STAT1, STAT2 and IRF9. Western blot of Py8119^Cas9^, Py8119^AhrKO_1^ and Py8119^AhrKO_2^ cells treated with DMSO, 10 µg/mL DMXAA, 100 nM RBN2397 or combination for 1–4 h, characterizing the IFN-I signalling pathway. 15 µg protein loaded. Representative image of *n* = 2

Since loss of AHR only affected the STING-induced IFN-I signalling in the Py8119 cells, we characterized the IFN-I signalling pathway in the Py8119^AhrKO^ clones versus Py8119^Cas9^ cells after 1–4 h treatment with DMXAA and/or RBN2397 by immunoblotting (Fig. 8, Supplementary Figure S13 and Supplementary Figure S14). Both AhrKO clones had lower constitutive and RBN2397-stabilized PARP7 protein levels compared with Cas9 cells, which is consistent with AHR’s role in regulating PARP7 levels. AHR protein levels were decreased in the Cas9 cells after 4 h with DMXAA, while no AHR was detected in the AhrKO clones. cGAS, pSTING (S365) and native STING expression patterns were similar between Cas9 and AhrKO cells. Similarly, *Ahr* loss did not affect pTBK1 (S172), or TBK1 expression patterns or levels. The ligand induced pIRF3 (S396) and IRF3 levels were similar between AhrKO clones and Cas9. IFNAR1 levels were notably lower in the AhrKO_2 compared with Cas9 cells and this was independent of the different treatments.

Py8119^AhrKO^ clones displayed similar pSTAT1 (Y701) pattern compared with Cas9 cells. We observed, however, increased levels of unphosphorylated STAT1, STAT2 and IRF9 in both AhrKO clones compared to Cas9, independently of the treatments. Although the pFOSL1 (S265) and FOSL1 patterns were similar between Cas9 and AhrKO cells were similar between AhrKO clones and Cas9 cells, the levels of both pFOSL1 (S265) and FOSL1 were lower in AhrKO_1. Together, these data suggest that loss of AHR increase IFN-I signalling in Py8119 cells through the unphosphorylated ISGF3 proteins.

### Loss or inhibition of AHR increases DMXAA-dependent RBN2397 sensitivity of Py230, but reduces sensitivity of Py8119 cells

**Fig. 9:**
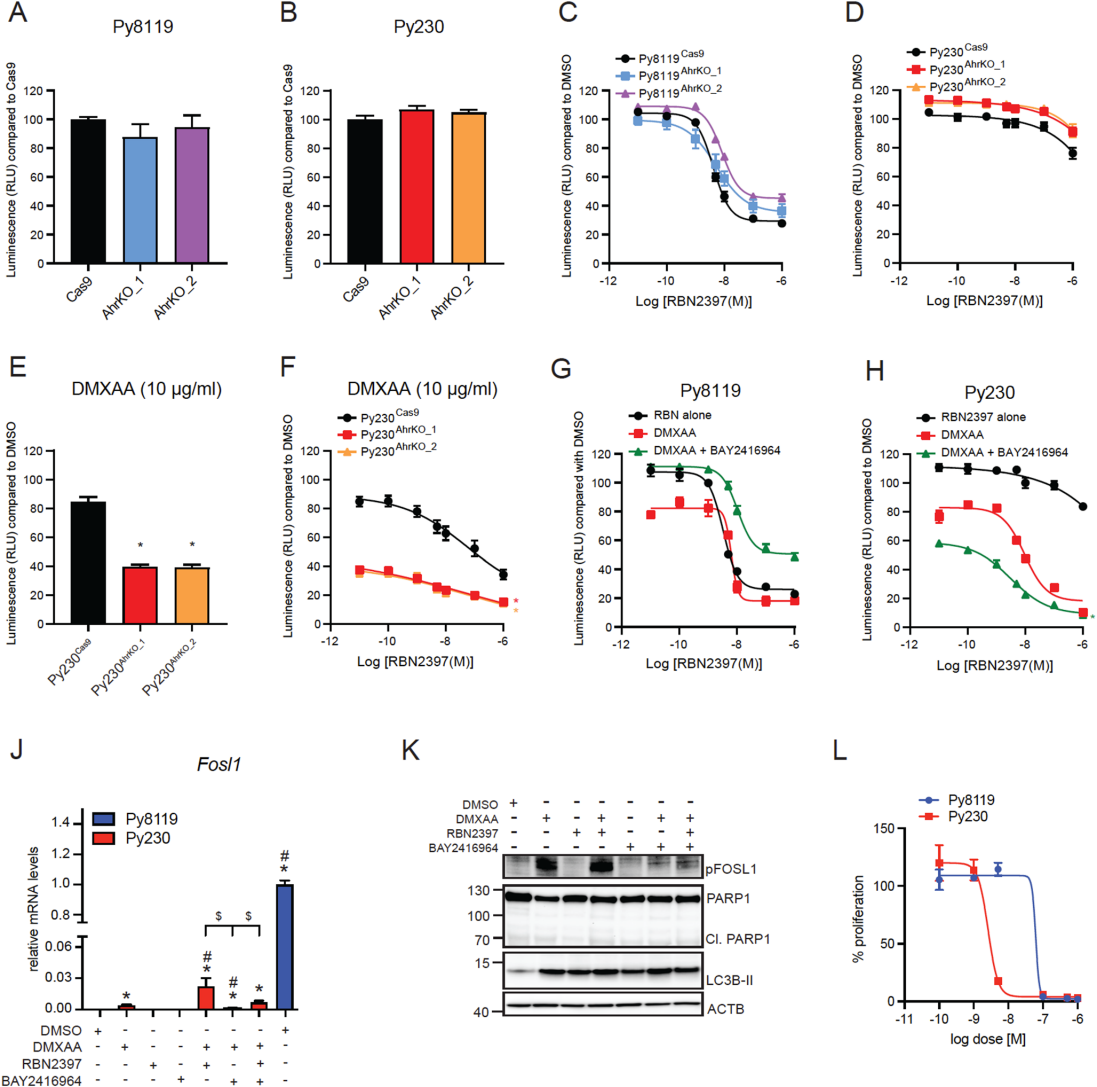
Loss of *Ahr* in combination with STING activation increased sensitivity of Py230 cells but reduced the sensitivity of Py8119 cells to RBN2397. Loss of *Ahr* did not affect proliferation of Py8119 (**A**) or Py230 (**B**) cells. (**C**) IC_50_ curve of Py8119^Cas9^, Py8119^AhrKO_1^ and Py8119^AhrKO_2^ cells treated with 0.01 to 1000 nM of RBN2397. (**D**) IC_50_ curve of Py230^Cas9^, Py230^AhrKO_1^ and Py230^AhrKO_2^ cells treated with 0.01 to 1000 nM of RBN2397, *n* = 8. (**E**) 10 µg/mL DMXAA decrease proliferation of Py230^AhrKO_1^ and Py230^AhrKO_2^ compared Py230^Cas9^ cells normalized to DMSO control for each cell line. (**F**) IC_50_ curve of Py230^Cas9^, Py230^AhrKO_1^ and Py230^AhrKO_2^ treated with 10 µg/mL DMXAA in combination 0.01 to 1000 nM of RBN2397, normalized to DMSO control for each cell line. IC_50_ curve of Py230 (**G**) and Py8119 (**H**) cells treated with fixed concentration of 10 µg/mL DMXAA and 1 µM BAY2416964 with 0.01 to 1000 nM RBN2397. RBN2397 alone and DMXAA + RBN2397 curves from Fig. 4C, G and J are included for comparison. *p < 0.05 compared with Py230^Cas9^ treated with DMXAA. (**J**) Fosl1 mRNA levels in Py230 cells treated for 48 h with DMSO, 10 µg/ml DMXAA, 100 nM RBN2397 and 1 µM BAY2416964 alone or in combination or DMSO treated Py8119 cells. *p < 0.05 compared with Py230 DMSO. # *p < 0.05 compared with Py230 DMXAA. $ *p* < 0.05 compared with Py230 DMXAA + RBN2397. (**K**) Western blot of Py230 cells treated DMSO, 10 µg/ml DMXAA, 100 nM RBN2397 and 1 µM BAY2416964 alone or in combination for 72 h. 30 µg protein loaded. Representative image of *n* = 2. (**L**) IC_50_ curve of Py8119 and Py230 cells treated with 0.1, to 1000 nM paclitaxel, *n* = 4

We then exposed Py8119 and Py230 AhrKO clones to increasing concentrations of RBN2397 to determine how loss of AHR affected sensitivity to PARP7 inhibition in both cell lines. In the absence of ligand treatment, the loss of *Ahr* did not significantly affect the proliferation of either cell line (Fig. 9A and B). We observed, however, a rightward shift in the inhibition curve and significant increase in IC_50_ values for RBN2397 in Py8119^AhrKO_2^ but not Py8119^AhrKO_1^ compared with Cas9 cells (Fig. 9C; Supplementary TableS1). Loss of *Ahr* did not affect the sensitivity of Py230 cells to RBN2397 (Fig. 9D), but treatment with DMXAA significantly decreased proliferation of both Py230 AhrKO clones (Fig. 9E). Cotreatment of DMXAA and RBN2397 also significantly decreased proliferation of Py230 AhrKO compared with Cas9 cells (Fig. 9F). BAY2416964 + DMXAA countered the antiproliferative effects of RBN2397 in Py8119 cells resulting in a rightward shift of the inhibition curve such that the bottom of the curve did not reach 50% and no absolute IC_50_ value could be calculated (Fig. 9G; Supplementary Table S1). In Py230 cells, BAY2416964 treatment in combination with DMXAA and RBN2397 resembled that of AhrKO cells resulting in decreased cell proliferation (Fig. 9H;Supplementary Table S2). Surprisingly, BAY2416964 treatment prevented DMXAA-dependent increases in *Fosl1* mRNA and pFOSL1 levels in Py230 cells (Fig. 9J and K). We observed increased LC3B-II, but no changes in cleaved PARP1 with single or combined treatments of DMXAA, RBN2397 and BAY2416964. These data suggest that the antiproliferative effects of DMXAA + RBN2397 in Py230 levels is due to increased or altered autophagy rather than apoptosis. Since a lack of apoptotic response in non-sensitive cell lines may explain their resistance to RBN2397, we tested the sensitivity of Py8119 and Py230 cells to paclitaxel (Fig. 9L). Py230 cells (IC_50_ = 3.2 ± 0.71 nM) are more sensitive than Py8119 cells (IC_50_ = 59 ± 2.2 nM) to the anti-proliferative effects of paclitaxel. These findings do not exclude that Py230 cells might be resistant to other inducers of apoptosis, but they confirm that they are sensitive to the antiproliferative effects of paclitaxel which is a known apoptotic activator.

## Discussion

PARP7 has gained considerable attention as a new target for antitumour immunity because its inhibition restores IFN-I signalling in cancer cells and results in durable tumour regression in preclinical mouse models [25, 41–43]. In addition, PARP7 functions as part of a negative feedback loop regulating AHR signalling. Both AHR and IFN signalling have been independently implicated in mediating the cell-autonomous effects of RBN2397. However, whether both pathways can function together to effect cancer cell sensitivity to PARP7 inhibition remain elusive. Here, we characterized the effects of RBN2397 on AHR and IFN-I signalling and cell proliferation in Py8119 and Py230 mammary cancer cell lines.

Consistent with PARP7 functioning as a negative regulator of AHR signalling, combining RBN2397 with the AHR agonist, FICZ, synergistically increased *Cyp1a1* compared with FICZ alone in both Py8119 and Py230 cells. The synergistic increase in *Cyp1a1* levels was higher in Py8119 cells because of the low increase in *Cyp1a1* by FICZ. These findings were supported by the FICZ- and FICZ + RBN2397-induced nuclear localization of AHR in both cell lines. RBN2397 increased FICZ-induced AHR recruitment to *Cyp1a1* in Py8119 but not in Py230 cells. This may be due to the time point chosen, for example earlier or perhaps later ligand treatments would reveal that RBN2397 has a similar effect on FICZ-induced AHR recruitment in Py230 cells. Collectively, this suggests that PARP7 more effectively inhibits AHR in Py8119, which would be consistent with the higher relative levels of PARP7 and increased *Cyp1a1* levels after 24 h RBN2397 treatment. The increase in *Cyp1a1* levels following prolonged RBN2397 treatment of Py8119 cells is most likely due to increased activity of cell culture media and endogenous derived AHR ligands after release of the inhibitor effects of PARP7 on AHR signalling. CYP1A1 levels have also been reported to be regulated by retinoic acid receptors and tyrosine kinase signal transduction pathways [44]. Thus, we cannot exclude that RBN2397 treatment might affect CYP1A1 levels independently of AHR. However, AHR is the predominant regular of CYP1A1 levels [45], which is further supported by the lack of *Cyp1a1* expression in AhrKO cells described here and in other studies [34, 46, 47].

In agreement with other reports, catalytic inhibition of PARP7 stabilized primarily nuclear PARP7 levels which were increased with AHR agonist treatment [28, 48]. These data support that RBN2397 causes stabilization and “nuclear trapping” of PARP7 that is enhanced with AHR agonist treatment. This outcome is comparable with olaparib-dependent stabilization and chromatin trapping of PARP1 and PARP2 [49]. PARP7 chromatin trapping has been suggested to contribute to growth inhibition of prostate cancer cells [28]. Additional studies, including chromatin immunoprecipitation studies will be important to understand how chromatin associated PARP7 affects cell proliferation and gene expression.

In line with that observed for AHR signalling, the high levels of PARP7 in Py8119 cells resulted in more effective suppression of IFN-I signalling compared with Py230 cells determined by the DMXAA + RBN2397 induced increases in IFN-I signalling compared with DMXAA alone. PARP7 has been shown to inhibit IFN-I through several different mechanisms [26, 50, 51]. In contrast to the viral induced IFN-I signalling [50], we found that TBK1 expression and phosphorylation levels were regulated by DMXAA and unaffected by RBN2397 in both cell lines. DMXAA-induced pIRF3 levels were increased by RBN2397 only in Py230 cells, which supports a role for PARP7 in regulating IRF3 [51]. However, these findings also suggest that the mechanisms of PARP7-mediated inhibition of IFN-I are cell line and context dependent. Despite AHR being reported to inhibit IFN-I signalling by facilitating the degradation of STING [31, 32], we did not observe any changes in STING levels in Py8119 AhrKO cells. AhrKO cells had lower PARP7 levels that would be consistent with AHR’s regulation of PARP7 expression. Reduced PARP7 protein levels would be expected to increase constitutive and inducible IFN-I signalling. Thus, the increased responsiveness of IFN-I signalling might be simply due to reduced PARP7 levels following loss of *Ahr*. The increased levels of ISGF3 proteins, STAT1, STAT2 and IRF9, is also in line with increased constitutive IFN-I and reduced PARP7 expression [26, 27, 52]. Since increased STAT1, STAT2 and IRF9 protein levels were DMXAA and not RBN2397-dependent in Py230 cells, ISGF3 protein levels are not necessarily dependent on PARP7.

Recently, sensitivity to the antiproliferative effect of RBN2397 in NCI-H1975 cells was shown to be dependent on FOSL1 in a mechanism involving IRF1 and IRF3 [18]. In their model, RBN2397 increases IRF1 levels that subsequently increased the interferon-stimulated gene, retinoic acid-inducible gene I (RIG-I) resulting in the phosphorylation of IRF3, which induces apoptosis. In line with this, RBN2397 sensitive Py8119 but not RBN2397 insensitive Py230 cells expressed FOSL1, and the FOSL1 levels in Py8119 cells were decreased with RBN2397. However, PARP7 expressing 4T1 and EO771 cells were not sensitive to PARP7 inhibition despite also expressing FOSL1 that was reduced by RBN2397. Our data show that high expression levels of PARP7 and FOSL1 do not necessarily predict sensitivity to RBN2397. The resistance of Py230 cells to RBN2397 could be due to a lack of a response to inducers of apoptosis; however, Py230 were more sensitive to paclitaxel than Py8119 cells. For Py8119 cells, the antiproliferative effect of PARP7i was independent of TBK1 activation and RBN2397 did not increase pIRF3 levels in the time points examined. RIG-I and STING pathways are interconnected and converge at TBK1 that directly phosphorylates IRF3 [53]. Although TBK1 and IkB kinase e are established IRF3 kinases, several other protein kinases, including DNA-PK, JNK and ERK1/2 have been reported to phosphorylate IRF3 [54–56]. Thus, it is possible that RBN2397-induced IRF3-dependent apoptosis occurs independently of TBK1-mediated phosphorylation of IRF3.

STING has been reported to regulate several pathways in an IFN-independent manner, including autophagy, metabolism homeostasis, DNA damage repair, senescence, cell proliferation and cell death [57]. For Py230 cells, activation of STING increased their sensitivity to RBN2397, by activating autophagy and not apoptosis. Although DMXAA treatment increased Fosl1 mRNA levels and pFOSL levels, the increases were not sufficient to detect changes in the levels of the apoptotic markers, cleaved PARP1 and CASP3. The reduced proliferation of Py230 cells was partially rescued with TBK inhibition, implicating IFN-I signalling in this effect. Additional studies engaging nucleic acid sensing PRRs using DNA or RNA viruses, polyI: C, or cGAMP will be needed to exclude that our results in Py230 cells are limited to STING activation.

We observed a smaller ∼70 kDa fragment of PARP1 that correlated with reduced proliferation in Py8119 and Py230 cells irrespective of whether apoptosis or autophagy was increased. A previous study reported that necrotic inducers, such as ethanol, can generate an active C-terminal PARP1 fragment at 72 kDa, or an inactive N-terminal fragment at 74 kDa with cathepsins B or G [58]. However, whether the observed ∼70 kDa band we observed represents N- or C-terminal fragment of PARP1 is not known. Additional studies are needed to determine the role, if any, that this PARP1 fragment plays in the antiproliferative effects of RBN2397 and if other pathways, such as necrosis are involved.

AHR agonist treatment has been shown to synergistic increase sensitivity to PARP7 inhibition in several different cancer cell lines, especially those insensitive RBN2397 [30]. However, AHR agonists had minimal to no effect on RBN2397 sensitivity in either cell line. Interestingly, *Ahr* loss or its inhibition in combination with DMXAA treatment significantly increased RBN2397 sensitivity of Py230 cells. The same treatments resulted in reduced RBN2397 sensitivity in Py8119 cells. This suggest that combination treatment of STING activation of RBN2397 could be pursued for cell lines that do not respond to RBN2397 alone. Although STING activation was the main driver of sensitivity to RBN2397 in Py230 cells, AHR inhibition with BAY2416964 and loss of AHR expression increased their sensitivity to RBN2397 + STING activation. In addition, BAY2416964 also prevented the DMXAA-induced increases in *Fosl1* levels, but how this further contributes to the reduced cell proliferation remains unknown. Regardless, these data suggest that that inhibiting AHR could be considered in cell lines that are sensitive to RBN2397 + STING activation.

Overall, our findings highlight the complexity of PARP7-AHR-IFN signalling interplay in regulating cancer cell proliferation but also support targeting this signalling axis as a potential cancer therapy. It will of course be necessary to confirm these in vitro findings in in vivo tumours models. Although PARP7 and AHR expression levels as well as an intact STING signalling pathway will be needed to target PARP7-AHR-IFN signalling for cancer treatment, identifying which cancer cells or tumours will respond to such interventions will still require the identification of robust biomarkers.

## Supplementary Information

Below is the link to the electronic supplementary material.

Supplementary Material 1

Supplementary Material 2

## Data Availability

Data is provided within the manuscript or supplementary information files.
