# Supplementary material for: PARP7 and aryl hydrocarbon receptor differentially regulate mammary cancer cell proliferation and STING-induced type I interferon signalling
Source: Cell Oncol (Dordr). 2025 Dec 23;49(1):3. doi: 10.1007/s13402-025-01150-w (PMC12727882; doi:10.1007/s13402-025-01150-w)
Supplement: Supplementary file 1 — Supplementary Material 1 [file 13402_2025_1150_MOESM1_ESM.pdf]

Supplementary Data

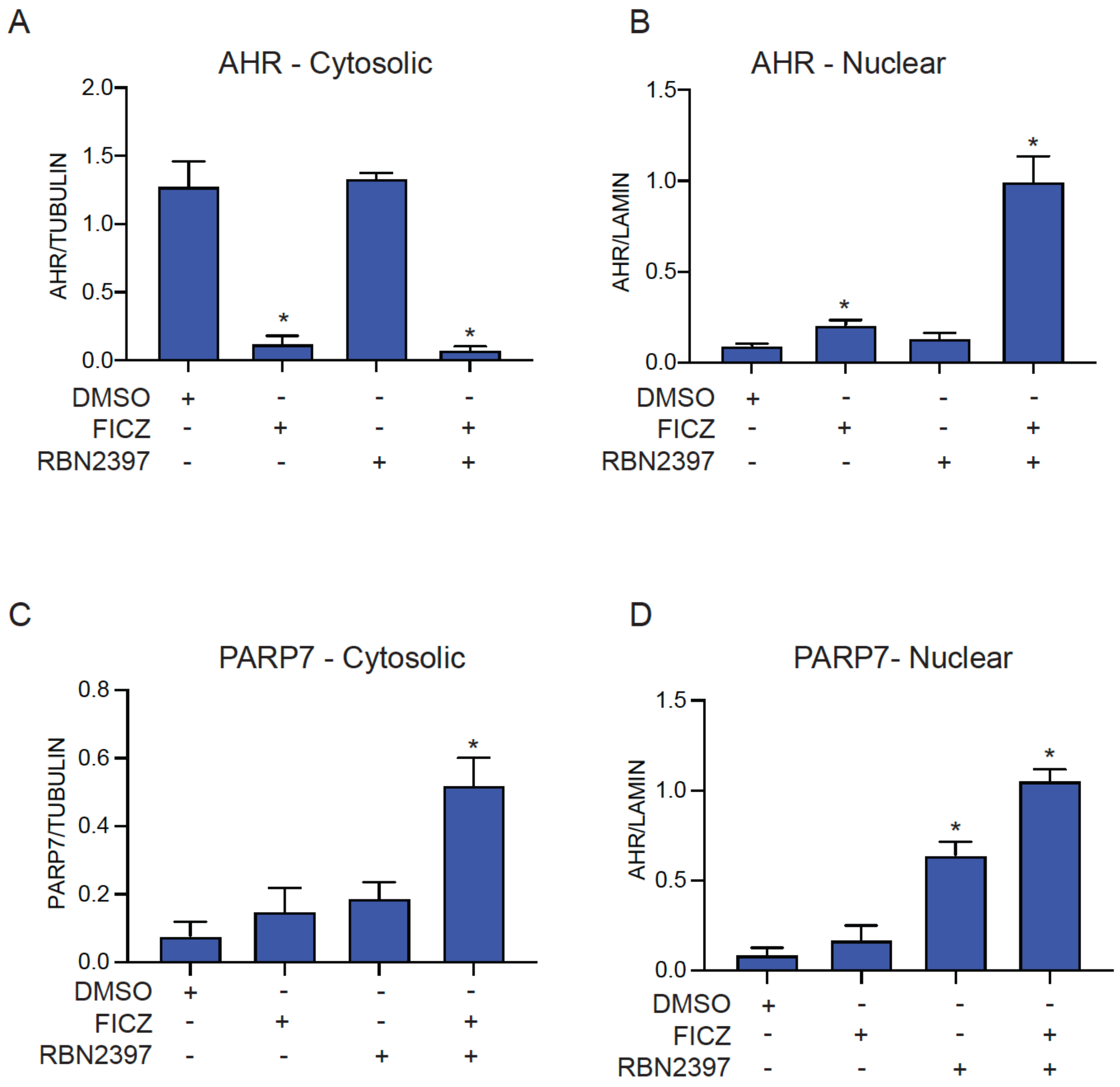

Supplementary Figure S1. Western blot quantification of data presented in Figure 2E for Py8119 cells. Cells were treated with DMSO, 10 nM FICZ and 100 nM RBN2397. Western blot images were quantified using ImageJ (National Institute of Health, Bethesda, MD, USA). * $p < 0.05$ compared with DMSO solvent control. Student's *t*-test. n=2.

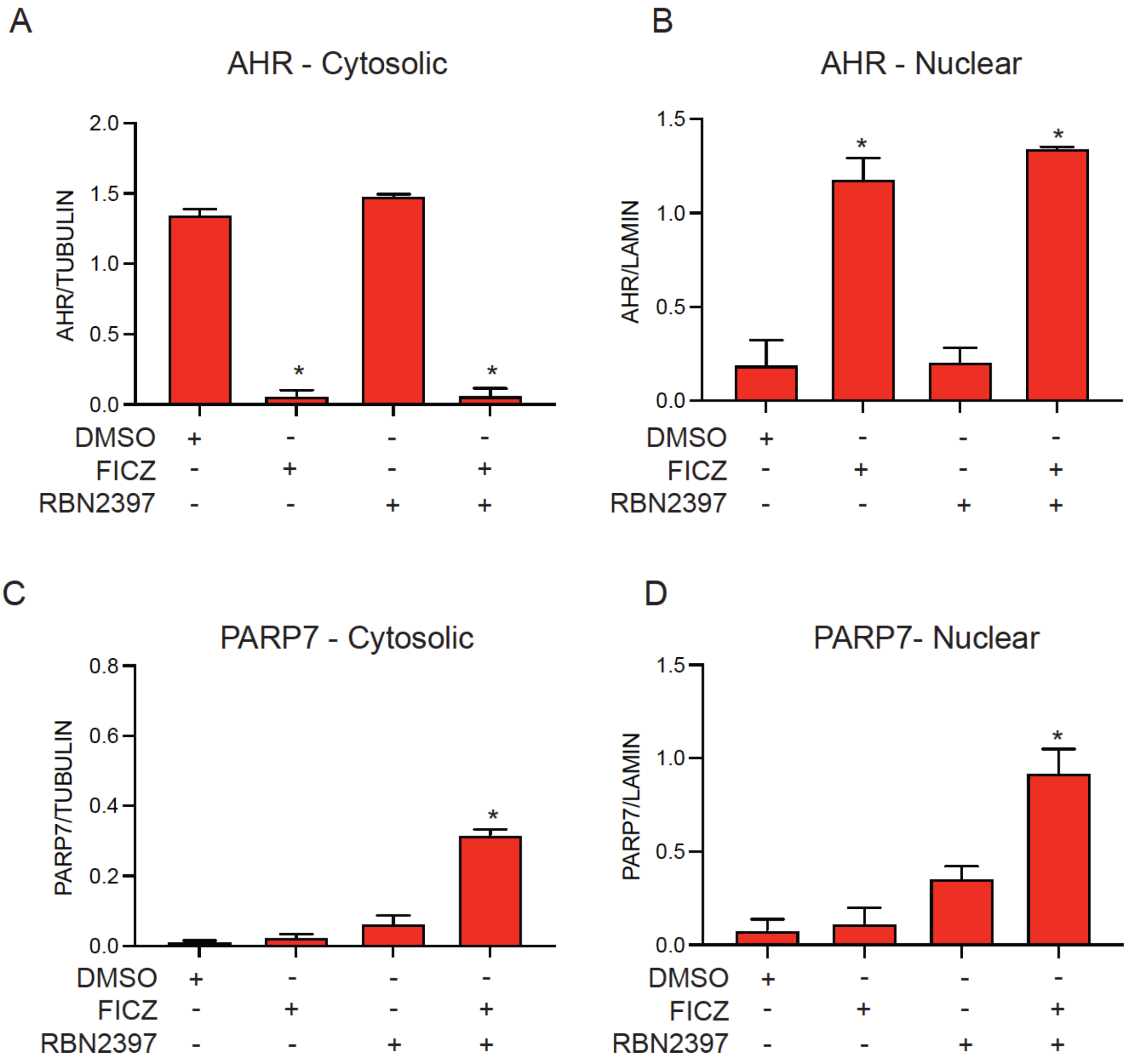

Supplementary Figure S2. Western blot quantification of data presented in Figure 2F for Py230 cells. Cells were treated with DMSO, 10 nM FICZ and 100 nM RBN2397. Western blot images were quantified using ImageJ (National Institute of Health, Bethesda, MD, USA). * $p < 0.05$ compared with DMSO solvent control. Student's *t*-test. *n*=2.

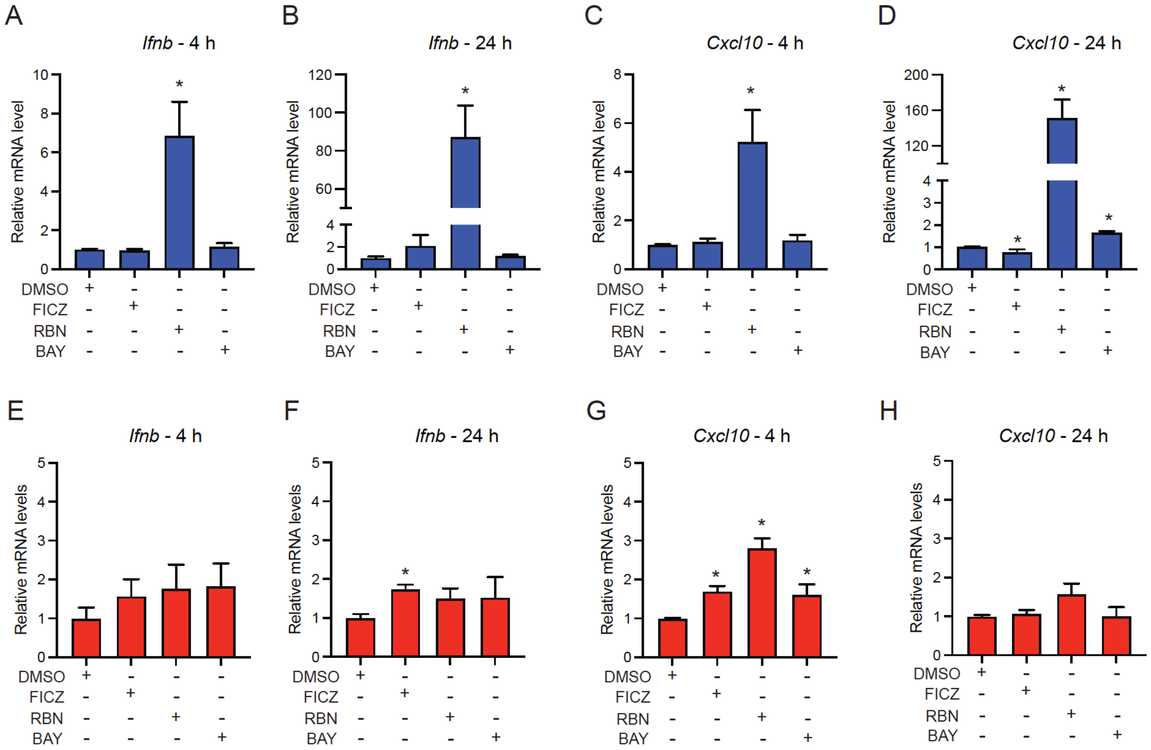

Supplementary Figure S3. (**A and B**) *Ifnb* and (**C and D**) *Cxcl10* mRNA levels in Py8119 cells. (**E and F**) *Ifnb* and (**G and H**) *Cxcl10* mRNA levels in Py230. Cells were treated with DMSO, 10 nM FICZ, 100 nM RBN2397 (RBN) or 1 μM BAY2416964 (BAY) for 4 h or 24 h. * $p < 0.05$ compared with cell line-matched DMSO. Student's *t*-test. *n*=3.

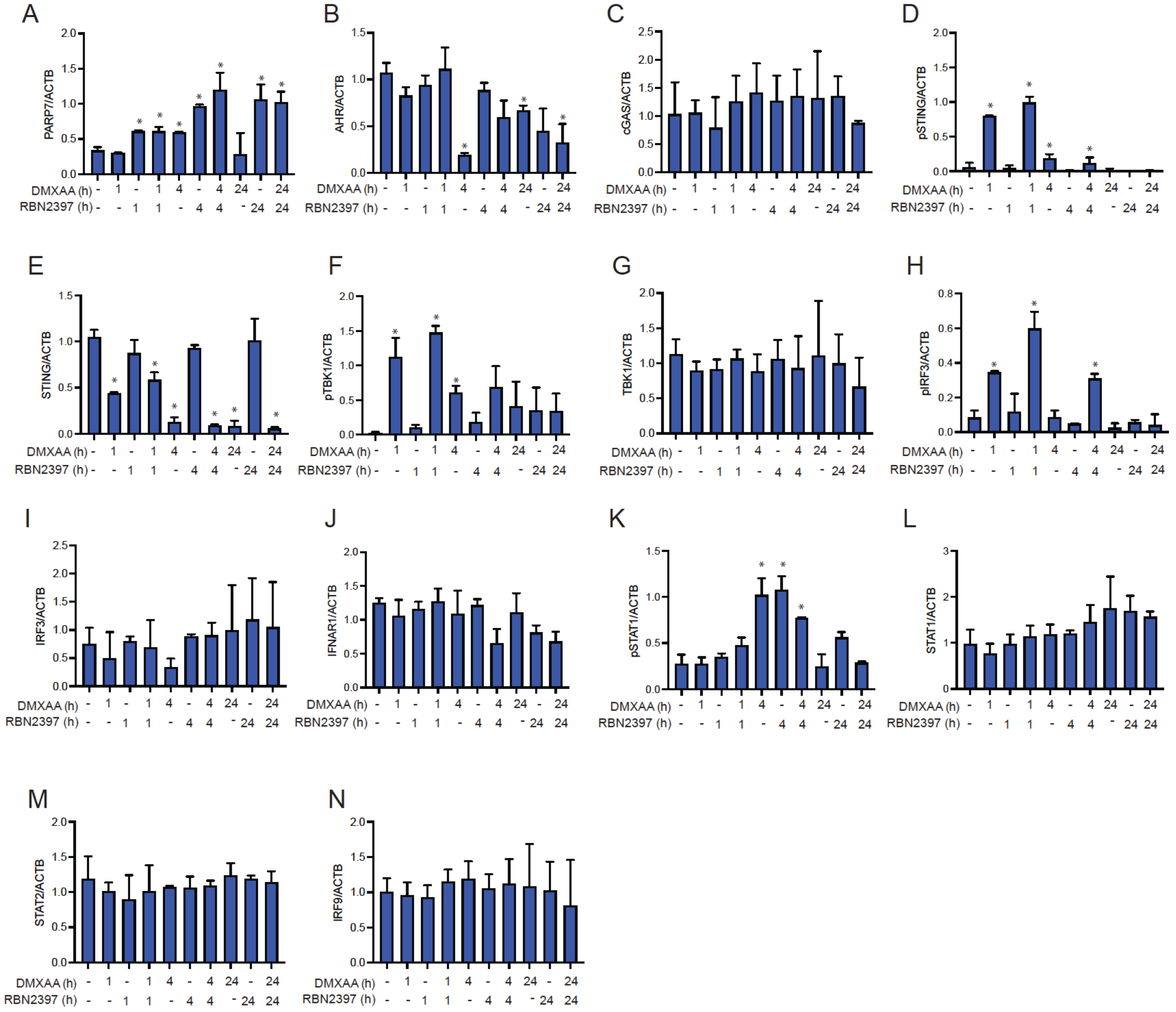

Supplementary Figure S4. Western blot quantification of data presented in Figure 3I for Py8119. Western blot images were quantified using ImageJ (National Institute of Health, Bethesda, MD, USA). * $p < 0.05$ compared with DMSO solvent control. Student's *t*-test. *n*=2.

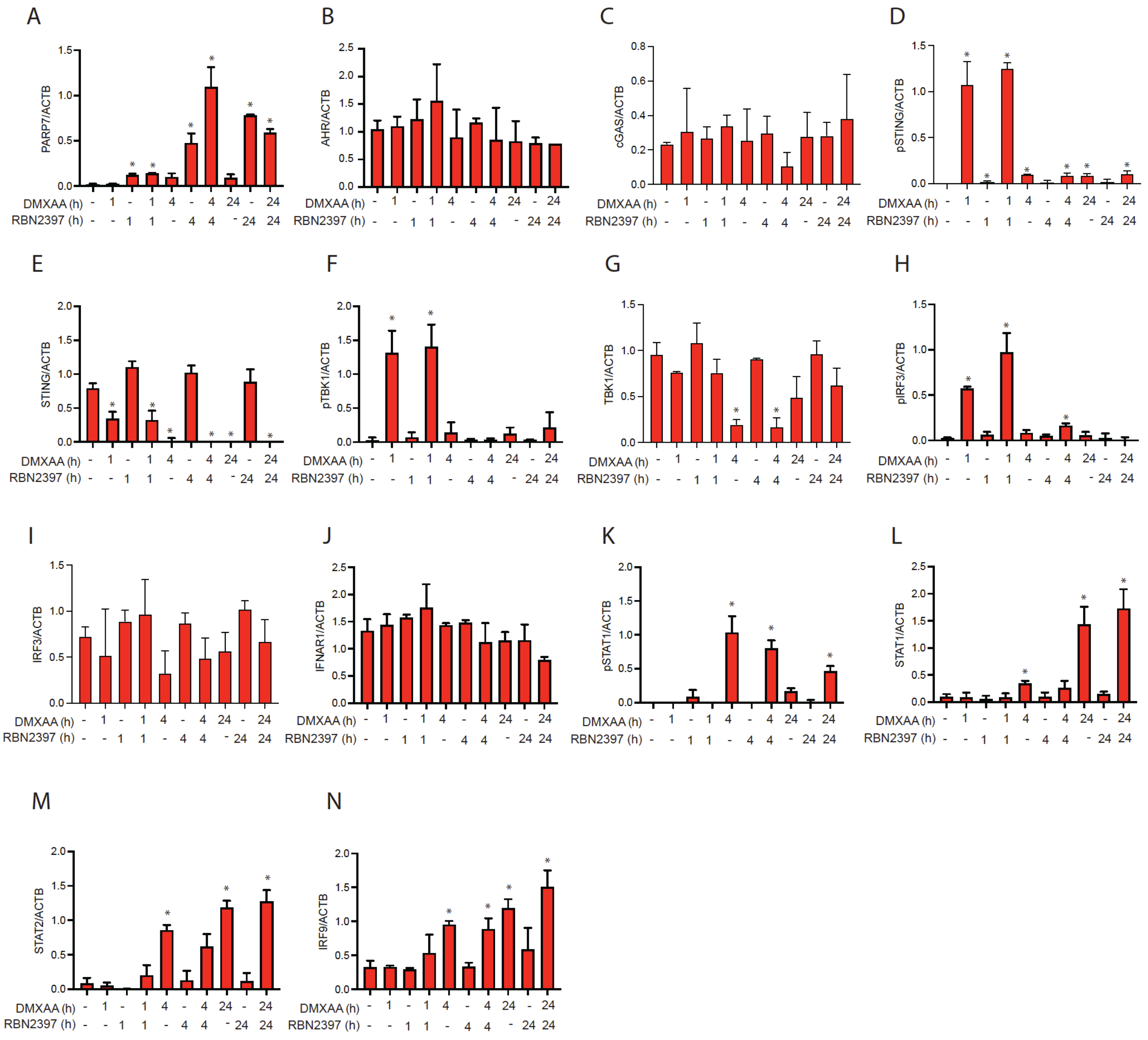

Supplementary Figure S5. Western blot quantification of data presented in Figure 3J for Py230 cells. Western blot images were quantified using ImageJ (National Institute of Health, Bethesda, MD, USA). * $p < 0.05$ compared with DMSO solvent control. Student's *t*-test. *n*=2.

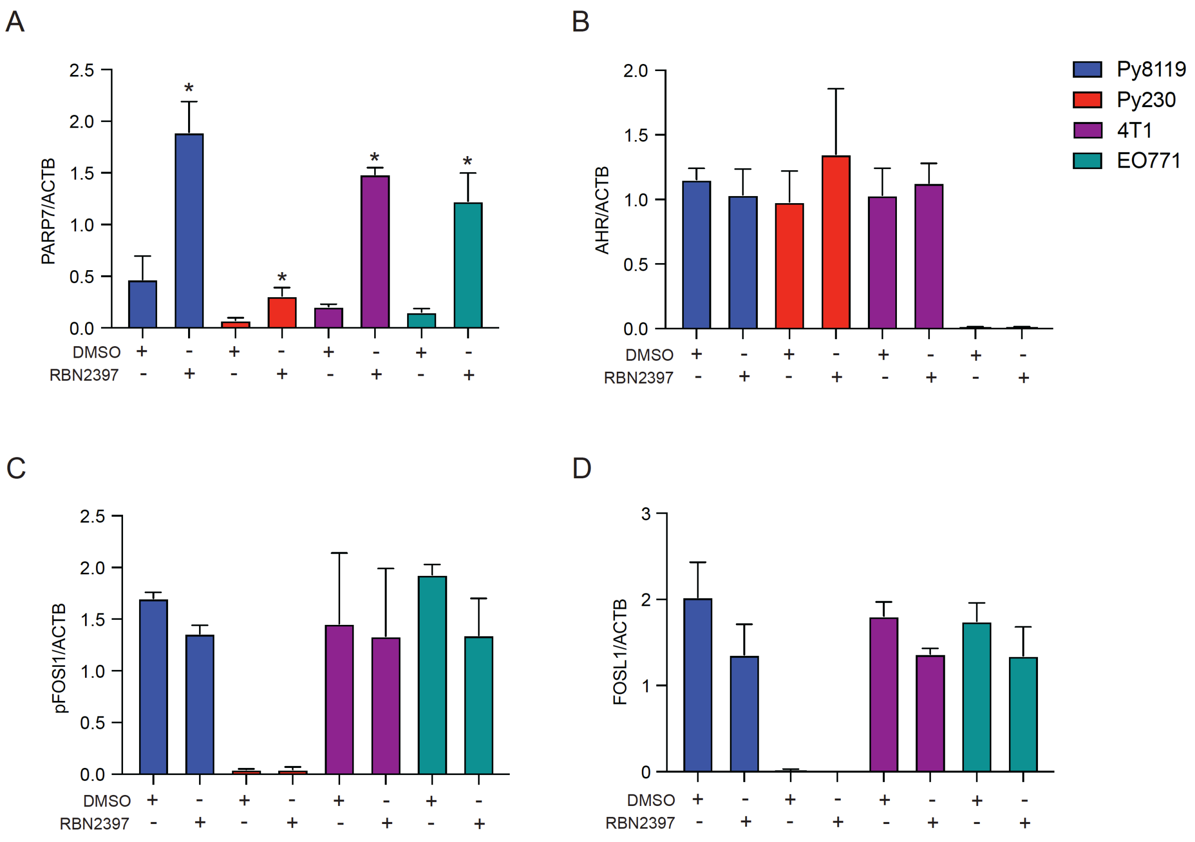

Supplementary Figure S6. Western blot quantification of the PARP7, AHR, pFOSL1 and FOSL levels in Py8119, Py230, 4T1 and EO771 cells. The western blot images presented in Figure 4E were quantified using ImageJ (National Institute of Health, Bethesda, MD, USA). Cells were treated with DMSO or 100 nM RBN2397 for 24 h. * $p < 0.05$ compared with cell line-matched DMSO solvent control. Student's *t*-test. n=3.

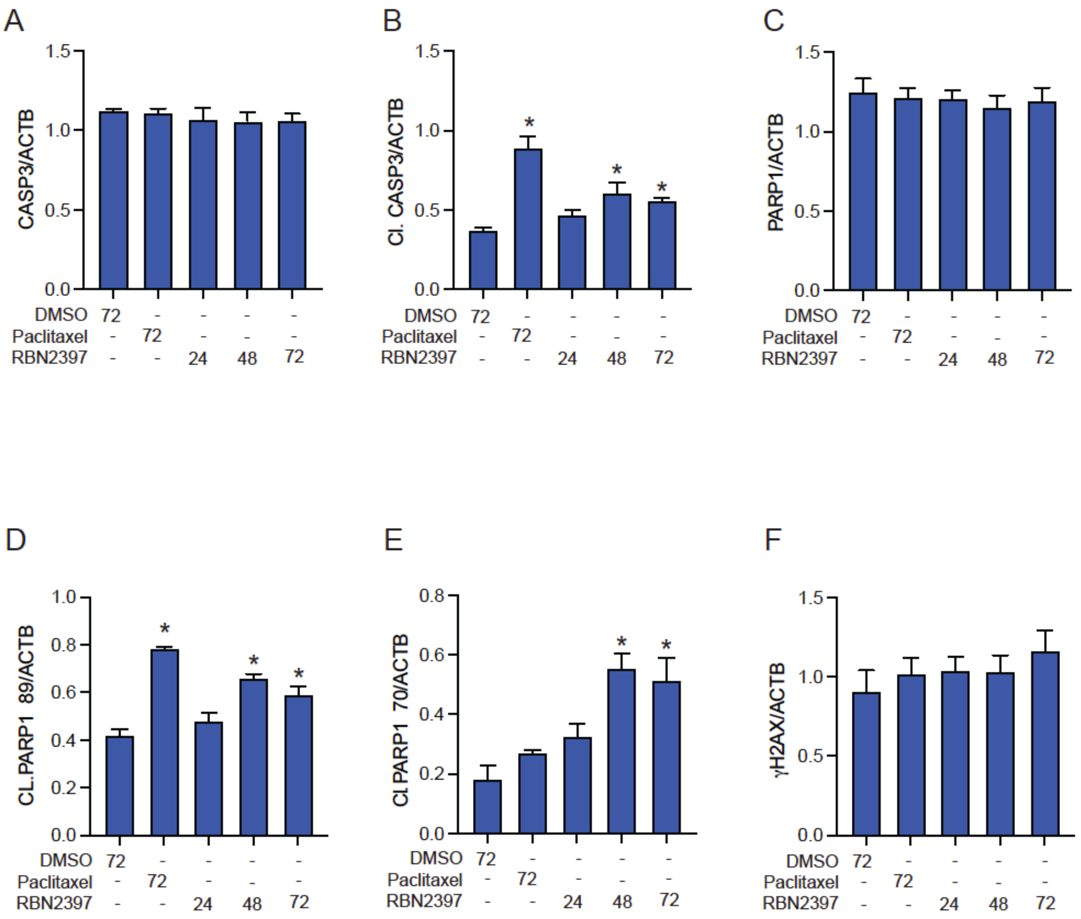

Supplementary Figure S7. Western blot quantification of data presented in Figure 4G. The western blot images presented in Figure 4G were quantified using ImageJ (National Institute of Health, Bethesda, MD, USA). Cells were treated with DMSO or 100 nM RBN2397 for 24 h. * $p < 0.05$ compared with cell line-matched DMSO solvent control. Student's *t*-test. *n*=3.

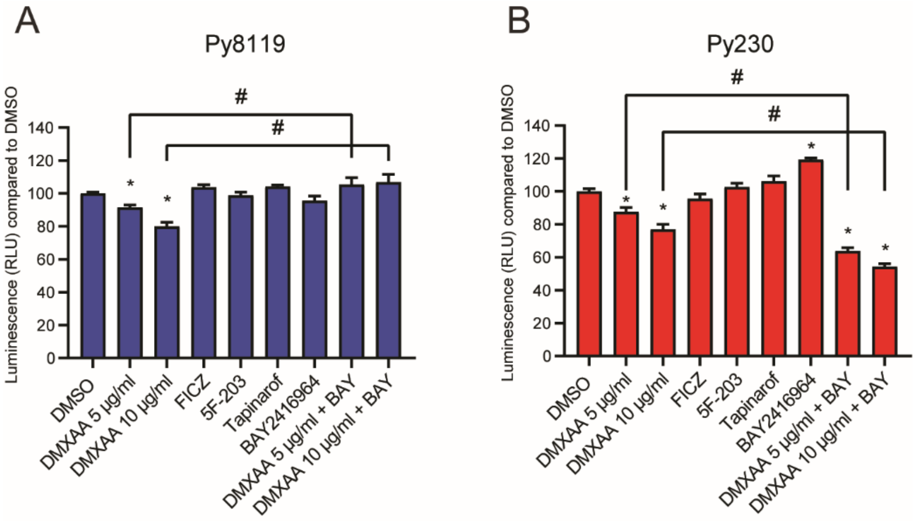

Supplementary Figure S8. Effect of DMXAA and AHR ligands on proliferation of Py8119 and Py230 cells. Proliferation of Py8119 (**A**) and Py230 (**B**) cells treated with 5 or 10 µg/ml DMXAA, 10 nM FICZ, 1 µM 5F-203, 1 µM tapinarof, or 1 µM BAY2416964, or combination of DMXAA and 1 µM BAY2416964 (BAY), *n*=2.

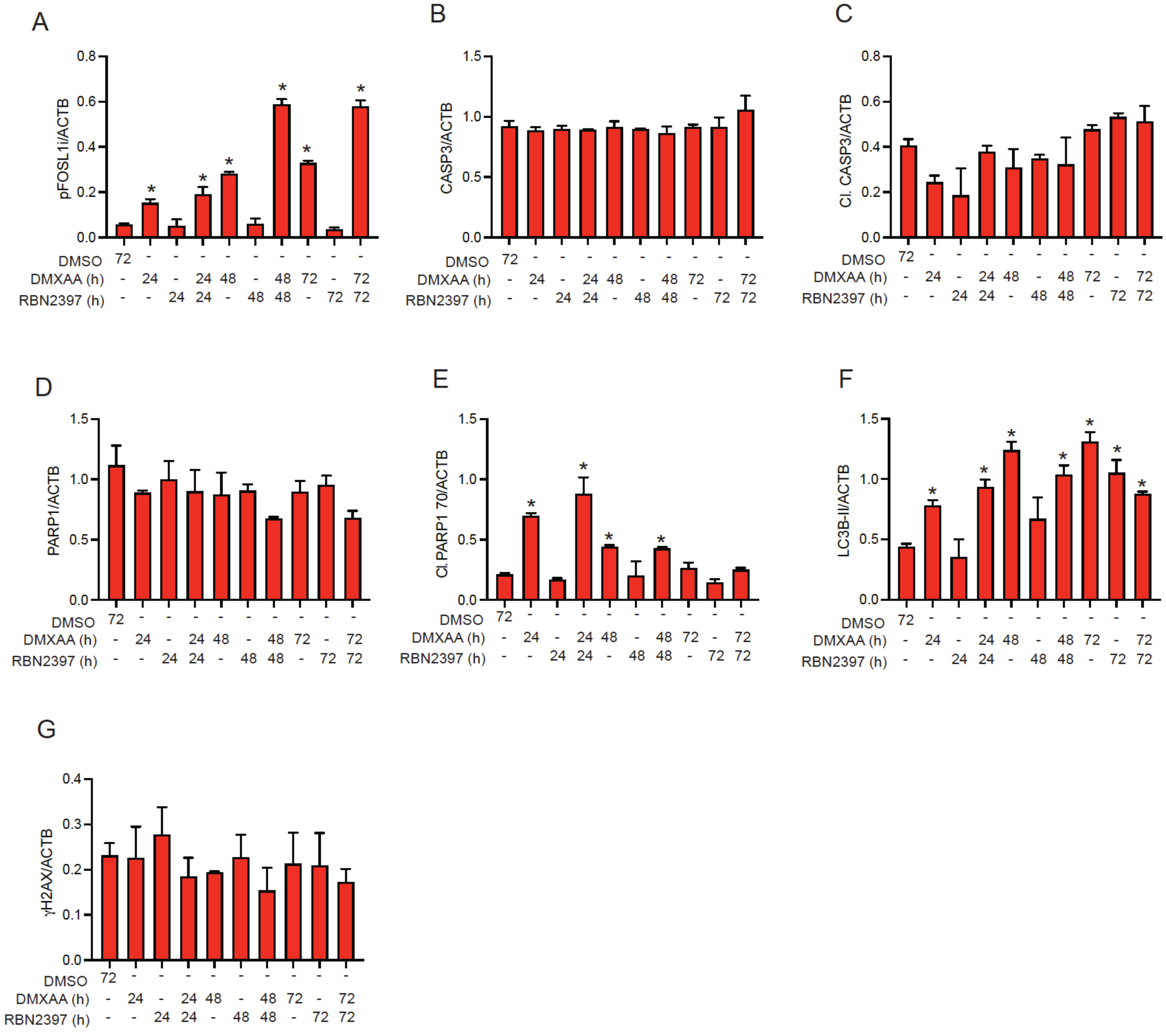

Supplementary Figure S9. Western blot quantification of data presented in Figure 5E. The western blot images presented in Figure 5E were quantified using ImageJ (National Institute of Health, Bethesda, MD, USA). Cells were treated with DMSO or 100 nM RBN2397 for 24 h. * $p < 0.05$ compared with cell line-matched DMSO solvent control. Student's *t*-test. n=2.

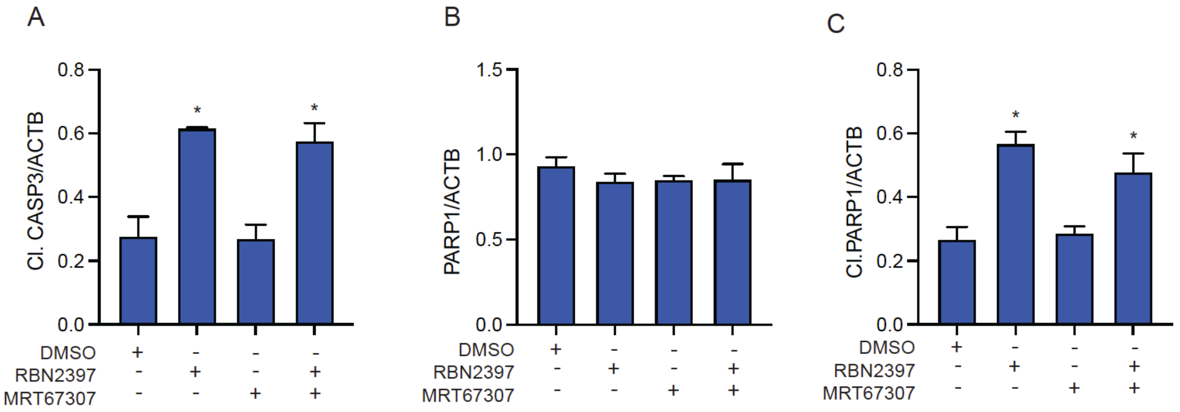

Supplementary Figure S10. Western blot quantification of data presented in Figure 5I (A) cleaved CASP3 (B) PARP1 and (C) Cleaved PARP1. The western blot images presented in Figure 5I were quantified using ImageJ (National Institute of Health, Bethesda, MD, USA). Cells were treated with DMSO, 100 nM RBN2397 and 1 µM MRT67307 for 24 h. * $p < 0.05$ compared with cell line-matched DMSO solvent control. Student's *t*-test. *n*=2.

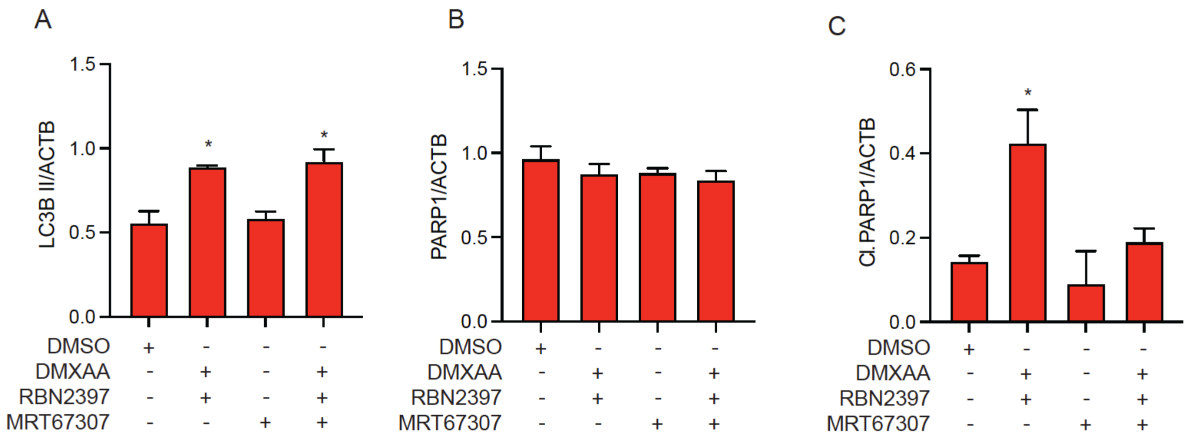

Supplementary Figure S11. Western blot quantification of data presented in Figure 5K (A) LC3BII (B) PARP1 and (C) Cleaved PARP1. The western blot images presented in Figure 5K were quantified using ImageJ (National Institute of Health, Bethesda, MD, USA). Cells were treated with DMSO, 10 µg/mL DMXAA, 100 nM RBN2397 and 1 µM MRT67307 for 24 h. * $p < 0.05$ compared with cell line-matched DMSO solvent control. Student's *t*-test. *n*=2.

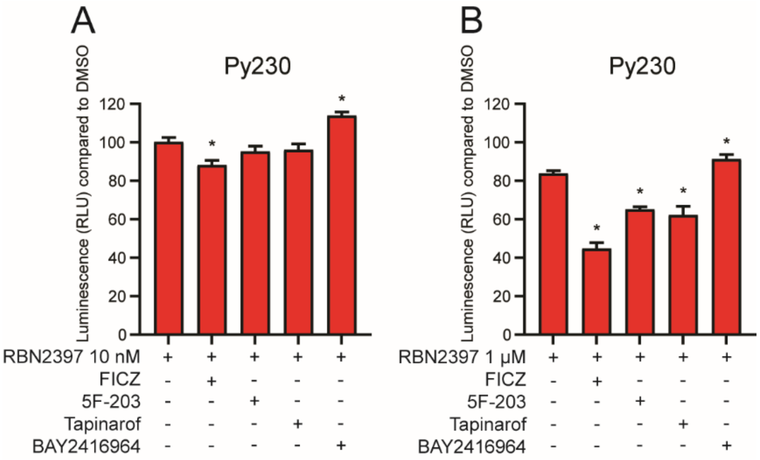

Supplementary Figure S12. Proliferation of Py230 cells with different concentrations of RBN2397 and AHR ligands. Proliferation of Py230 cells treated with 10 nM RBN2397 (**A**) or 1 µM RBN2397 (**B**) alone or in combination with 10 nM FICZ, 1 µM 5F-203, 1 µM tapinarof, or 1 µM BAY2416964. * Significance from RBN2397, tested with Student's *t*-test, *n*=2.

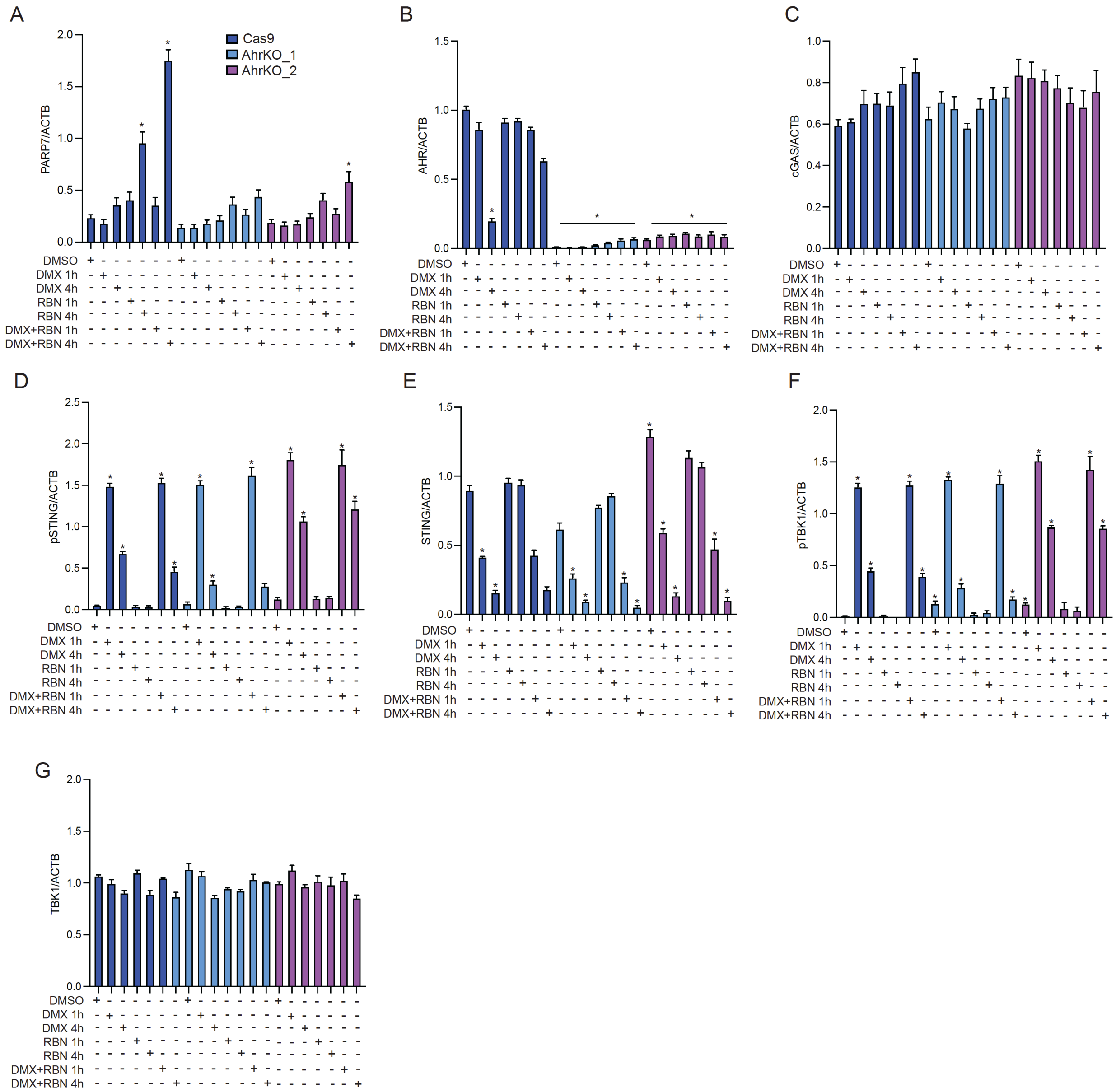

Supplementary Figure S13. Western blot quantification of data presented in Figure 8 of the proteins indicated. The western blot images presented in Figure 8 were quantified using ImageJ (National Institute of Health, Bethesda, MD, USA). Cells were treated with DMSO, 10 µg/mL DMXAA (DMX), 100 nM RBN2397 (RBN) and their combination for 1 h and 4 h. * $p < 0.05$ compared with cell line-matched DMSO of Cas9. Student's *t*-test. *n*=2.

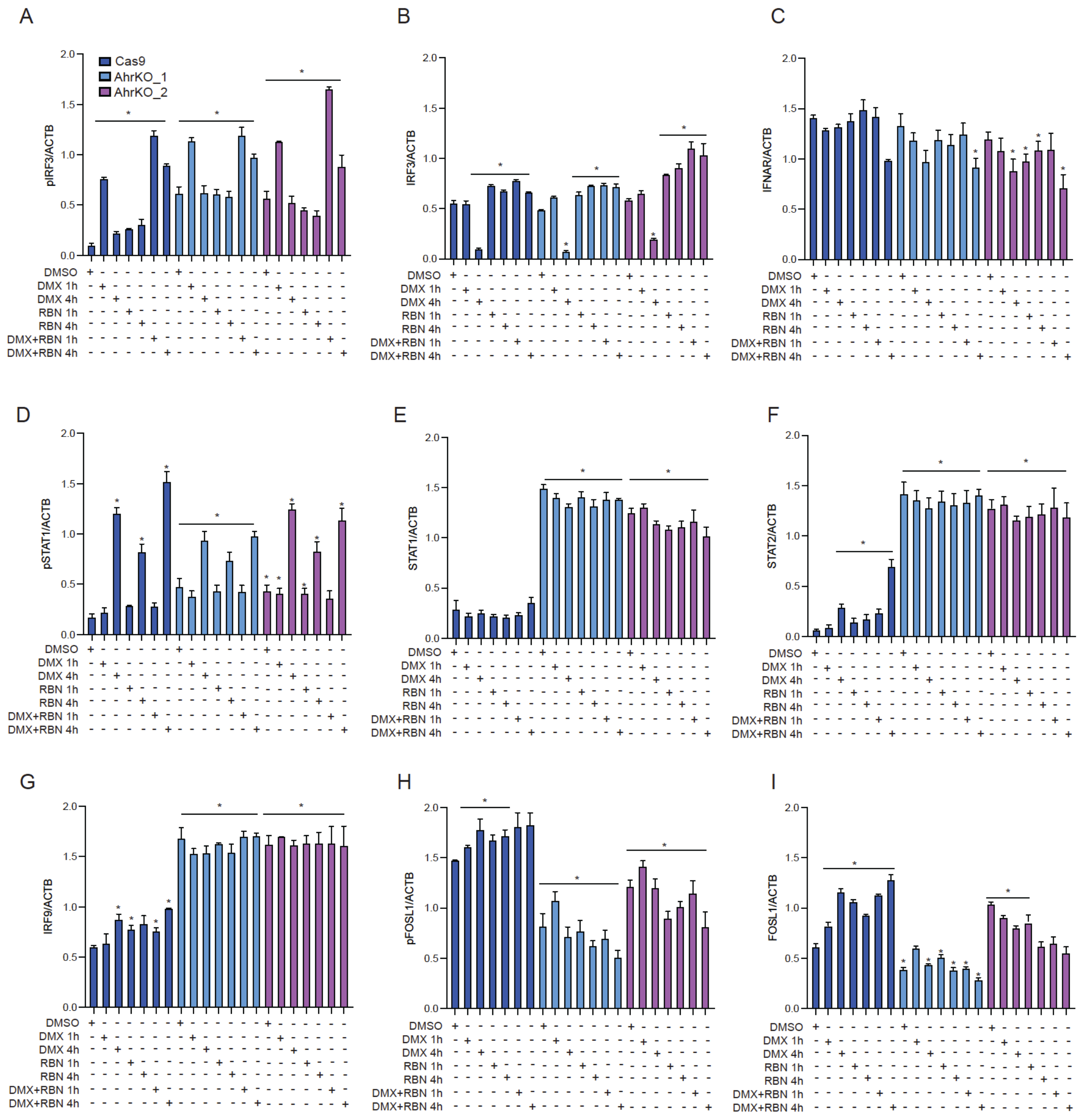

Supplementary Figure S14. Western blot quantification of data presented in Figure 8 of the proteins indicated. The western blot images presented in Figure 8 were quantified using ImageJ (National Institute of Health, Bethesda, MD, USA). Cells were treated with DMSO, 10 µg/mL DMXAA (DMX), 100 nM RBN2397 (RBN) and their combination for 1 h and 4 h. * $p < 0.05$ compared with cell line-matched DMSO of Cas9. Student's *t*-test. n=2.

Supplementary Table S1. IC_{50} summary of Py8119 cells treated with compounds in combination with increasing concentrations of RBN2397.

Cell line	Compound with increasing concentration of RBN2397	IC_{50} (M)	p-value compared to RBN2397	Significance
Py8119	RBN2397	$5.6\times10^{-9} \pm 5.2\times10^{-10}$		
	DMXAA (5 µg/mL)	$3.5\times10^{-8} \pm 5.1\times10^{-8}$	0.1664	No
	DMXAA (10 µg/mL)	$4.7\times10^{-9} \pm 2.6\times10^{-9}$	0.2887	No
	FICZ (10 nM)	$4.6\times10^{-9} \pm 1.9\times10^{-9}$	0.2165	No
	5F-203 (1 µM)	$1.0\times10^{-8} \pm 6.6\times10^{-9}$	0.135	No
	Tapinarof (1 µM)	$1.0\times10^{-8} \pm 5.4\times10^{-9}$	0.1010	No
	BAY2416964 (1 µM)	$2.0\times10^{-8} \pm 8.2\times10^{-9}$	0.0042	Yes
	DMXAA (10 µg/mL) + BAY2416964 (1 µM)[a]	-	n.d.	n.d.
$Py8119^{Cas9}$	RBN2397	$4.5\times10^{-9} \pm 9.4\times10^{-10}$		
$Py8119^{AhrKO\_1}$	RBN2397	$5.8\times10^{-9} \pm 3.1\times10^{-9}$	0.2261[b]	No
$Py8119^{AhrKO\_2}$	RBN2397	$8.5\times10^{-9} \pm 1.9\times10^{-9}$	<0.0001[b]	Yes

[a] Bottom of the curve did not reach 50% inhibition. Not determined, n.d.

[b] Compared to $Py8119^{Cas9}$ with RBN2397.

Supplementary Table S2. IC_{50} summary of Py230 cells treated with compounds in combination with increasing concentrations of RBN2397.

Cell line	Compound with increasing concentration of RBN2397	IC_{50} (M)	p-value compared to DMXAA	Significance
Py230	DMXAA (10 µg/mL)	$9.7 \times 10^{-9} \pm 1.9\times10^{-9}$		
	DMXAA (10 µg/mL) + BAY2416964 (1 µM)	$4.5 \times 10^{-10} \pm 2.5\times10^{-10}$	0.0002	Yes

Supplementary Table S3. Summary of indels identified in Py8119^{AhrKO} and Py230^{AhrKO} clones upon DNA sequencing of exon 2 of *Ahr*.

Cell line	Clone	Indel (no. with mutation/total sequenced)	Premature stop codon
Py8119	AhrKO_1	Missing four nucleotides (23/24)	Yes
		Missing 404 nucleotides (1/24)	
	AhrKO_2	Missing eight nucleotides (6/8)	Yes
		Missing 26 nucleotides (2/8)	
Py230	AhrKO_1	Missing one nucleotide (10/17)	Yes
		Missing two nucleotides (6/17)	
		Insert 296 nucleotides (1/17)	
	AhrKO_2	Missing one nucleotide (8/17)	Yes
		Missing two nucleotides (7/17)	
		Insert one nucleotide (2/17)	
