# Supplementary material for: PARP7 and aryl hydrocarbon receptor differentially regulate mammary cancer cell proliferation and STING-induced type I interferon signalling
Source: Cell Oncol (Dordr). 2025 Dec 23;49(1):3. doi: 10.1007/s13402-025-01150-w (PMC12727882; doi:10.1007/s13402-025-01150-w)

Uncropped images of all gels and blots.

Uncropped western blots from Figure 1A

Figure 1A

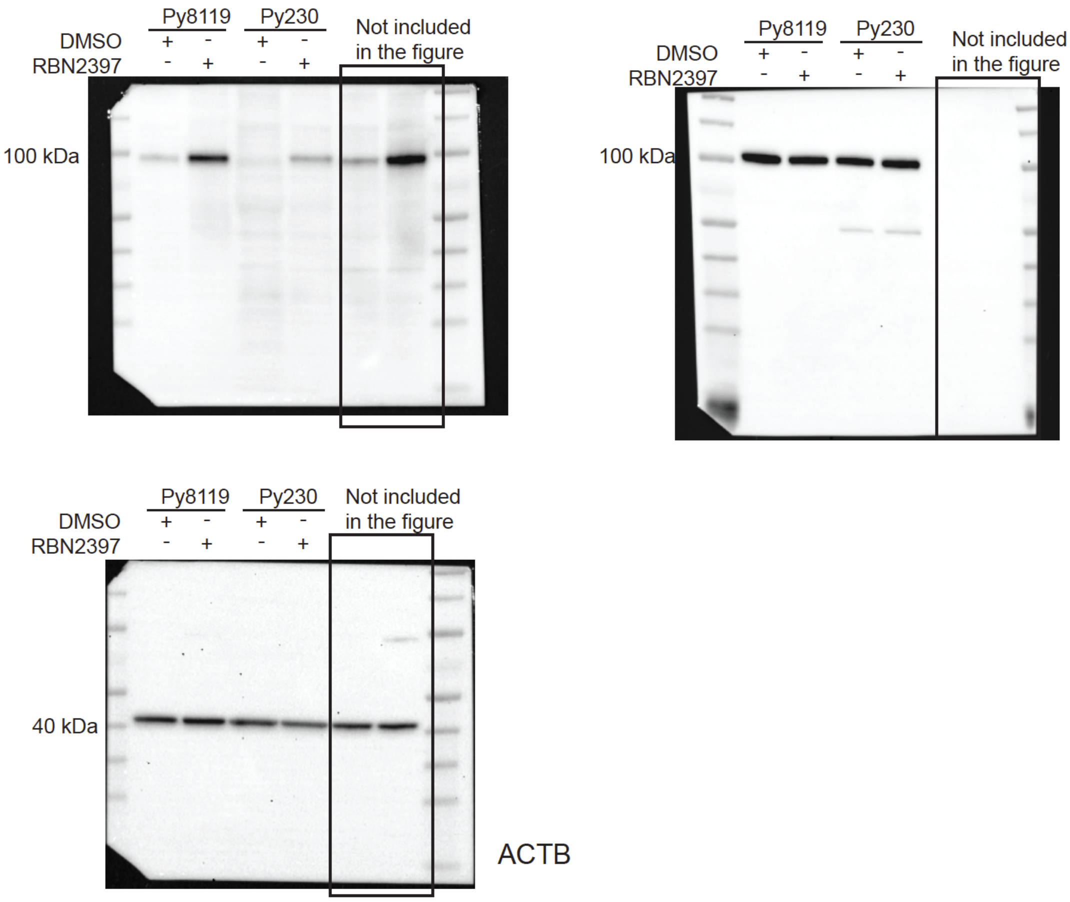

Uncropped western blots from Figure 2E

Figure 2E

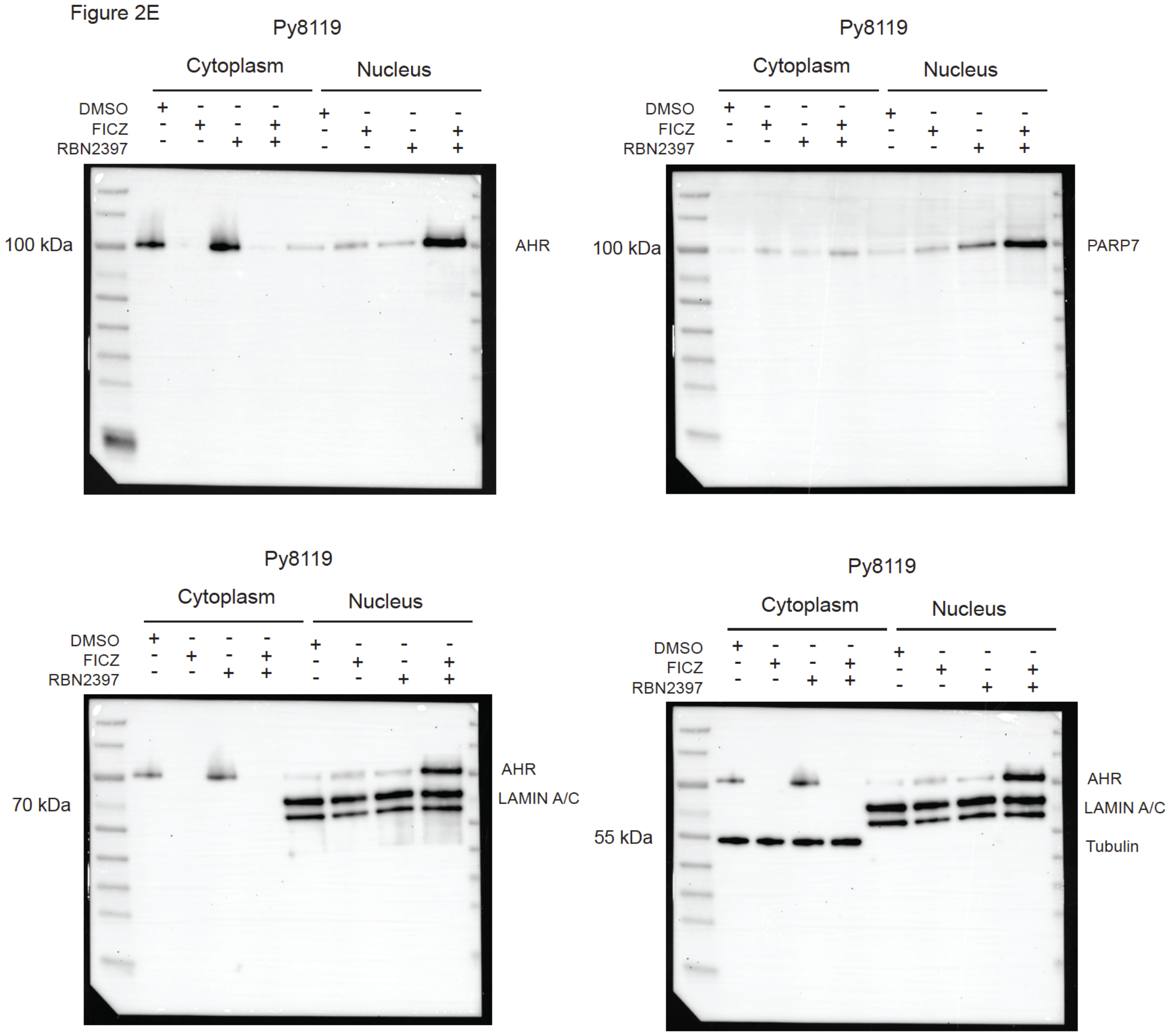

Uncropped western blots from Figure 2F

Figure 2F

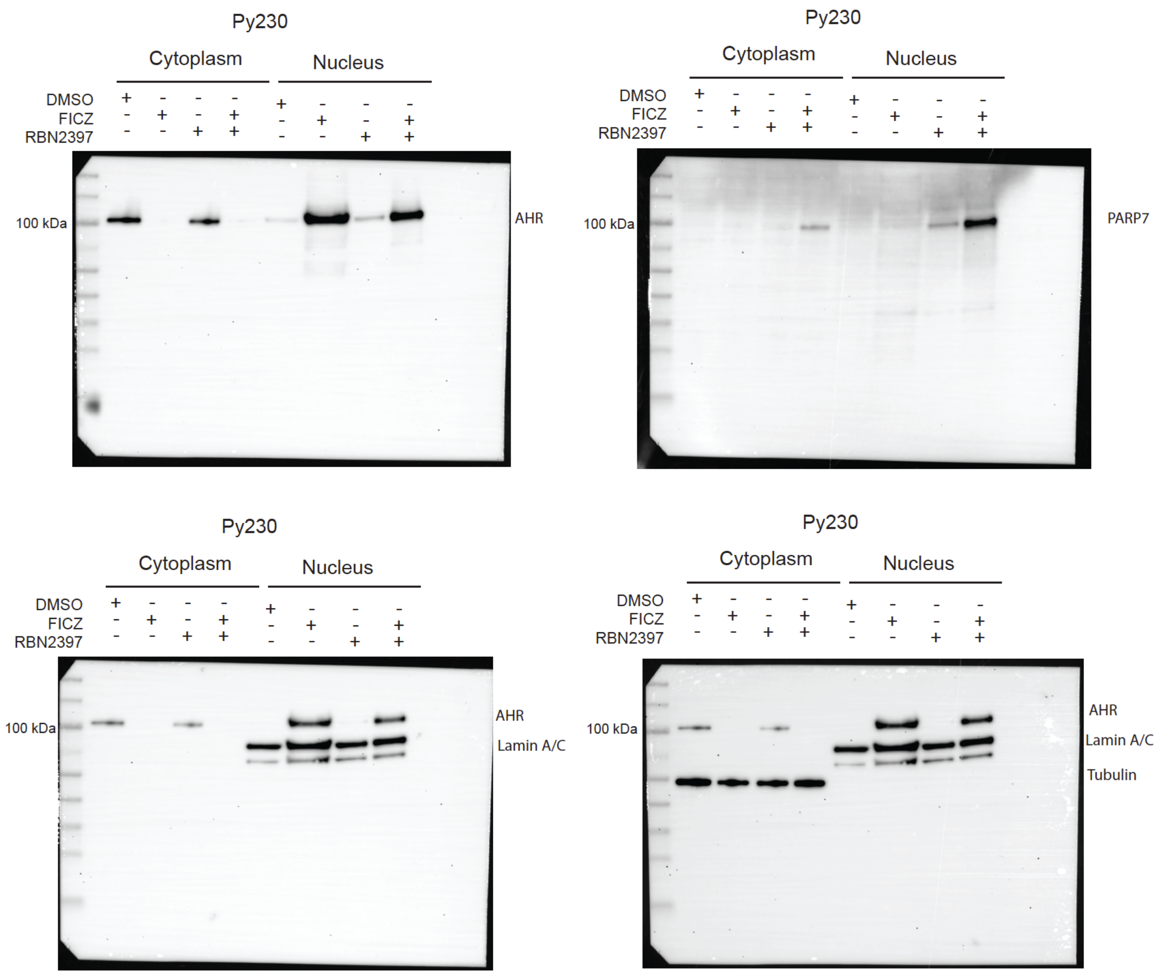

Uncropped western blots from Figure 3I and 3J

Figure 3I and 3J

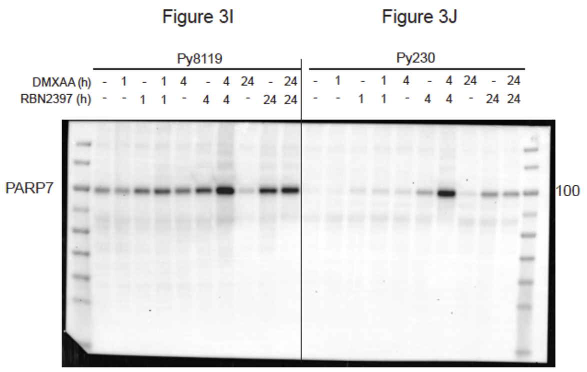

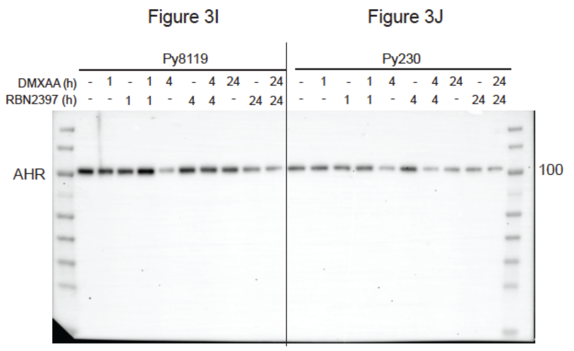

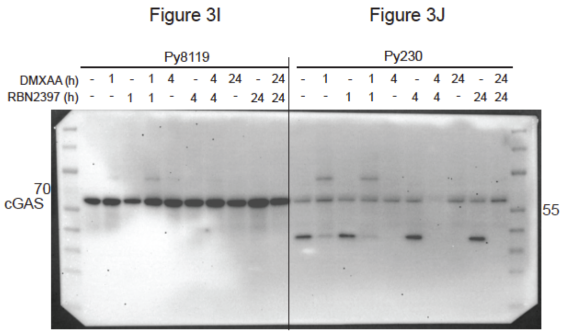

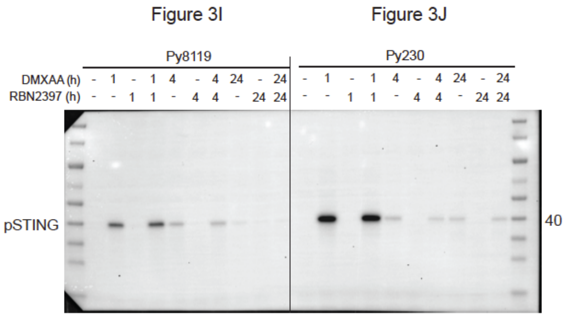

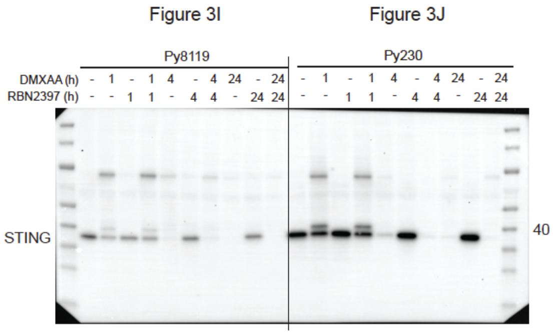

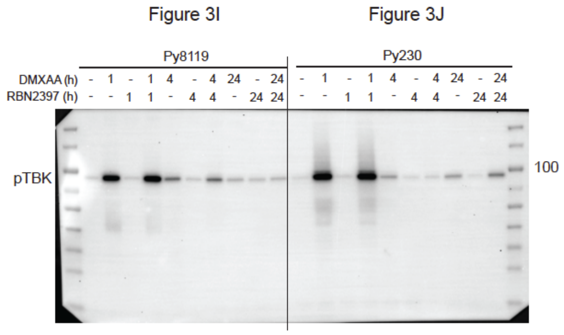

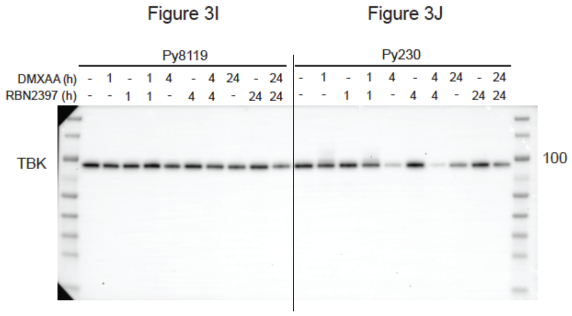

Uncropped western blots from Figure 3I and 3J continued

Figure 3I and 3J

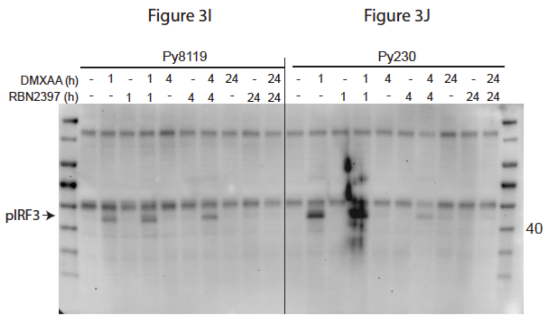

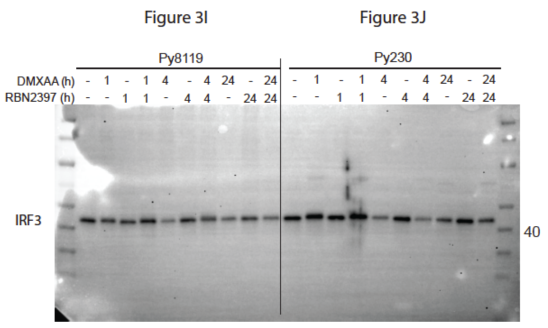

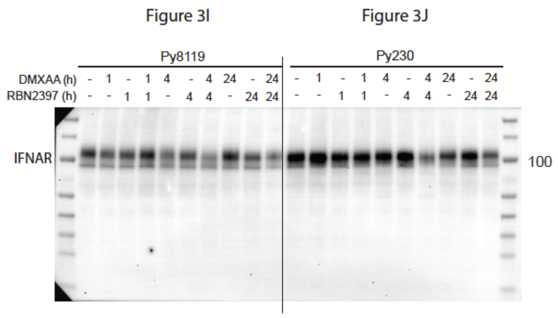

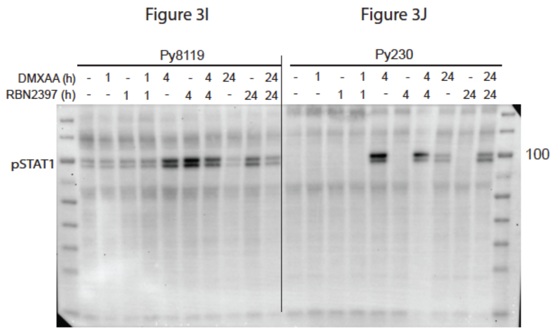

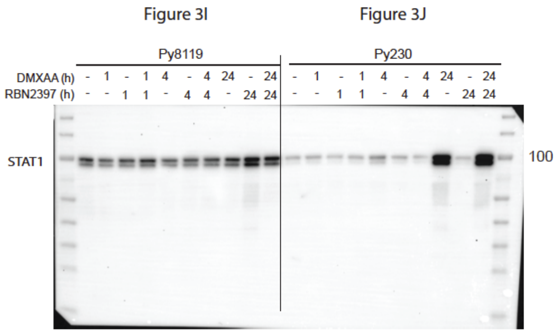

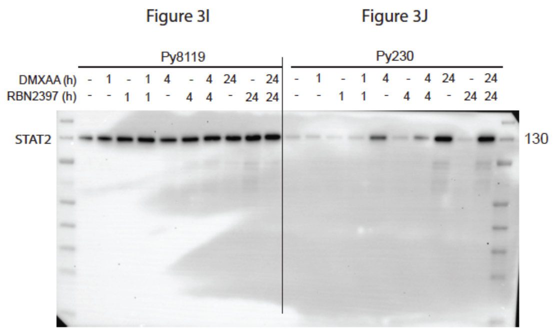

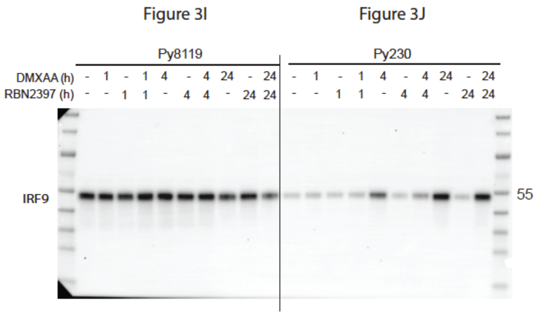

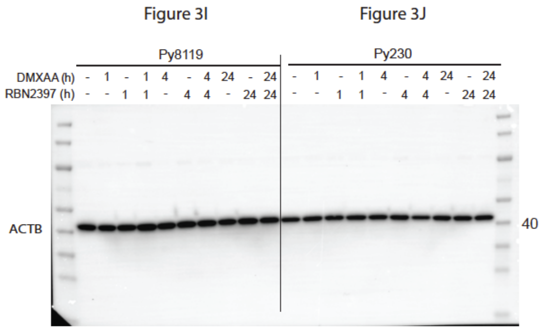

Uncropped western blots from Figure 4E

Figure 4E

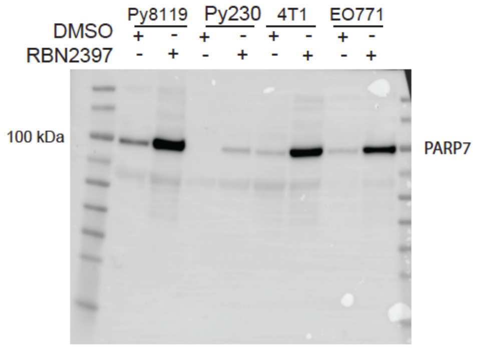

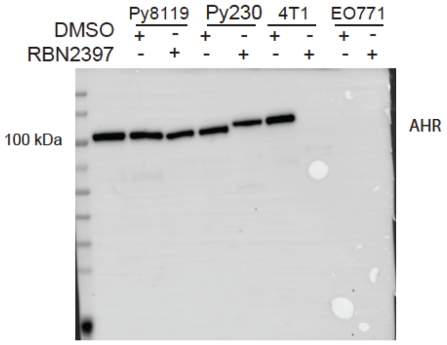

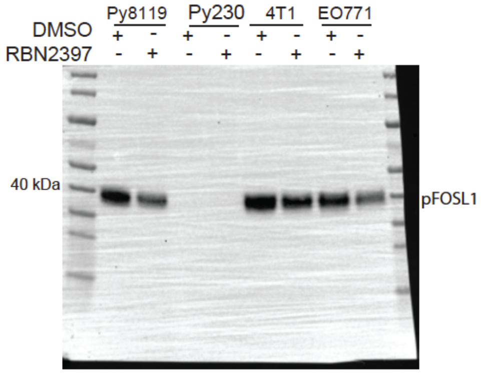

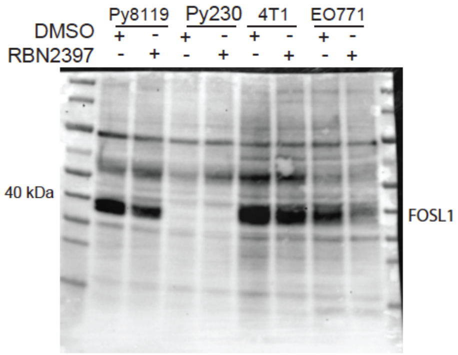

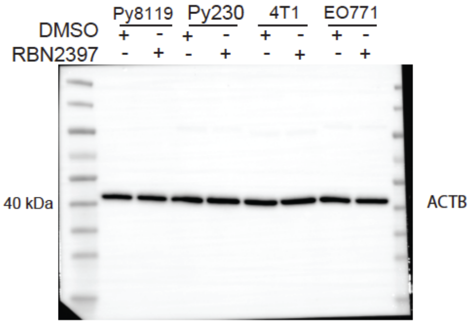

Uncropped western blots from Figure 4G

Figure 4G

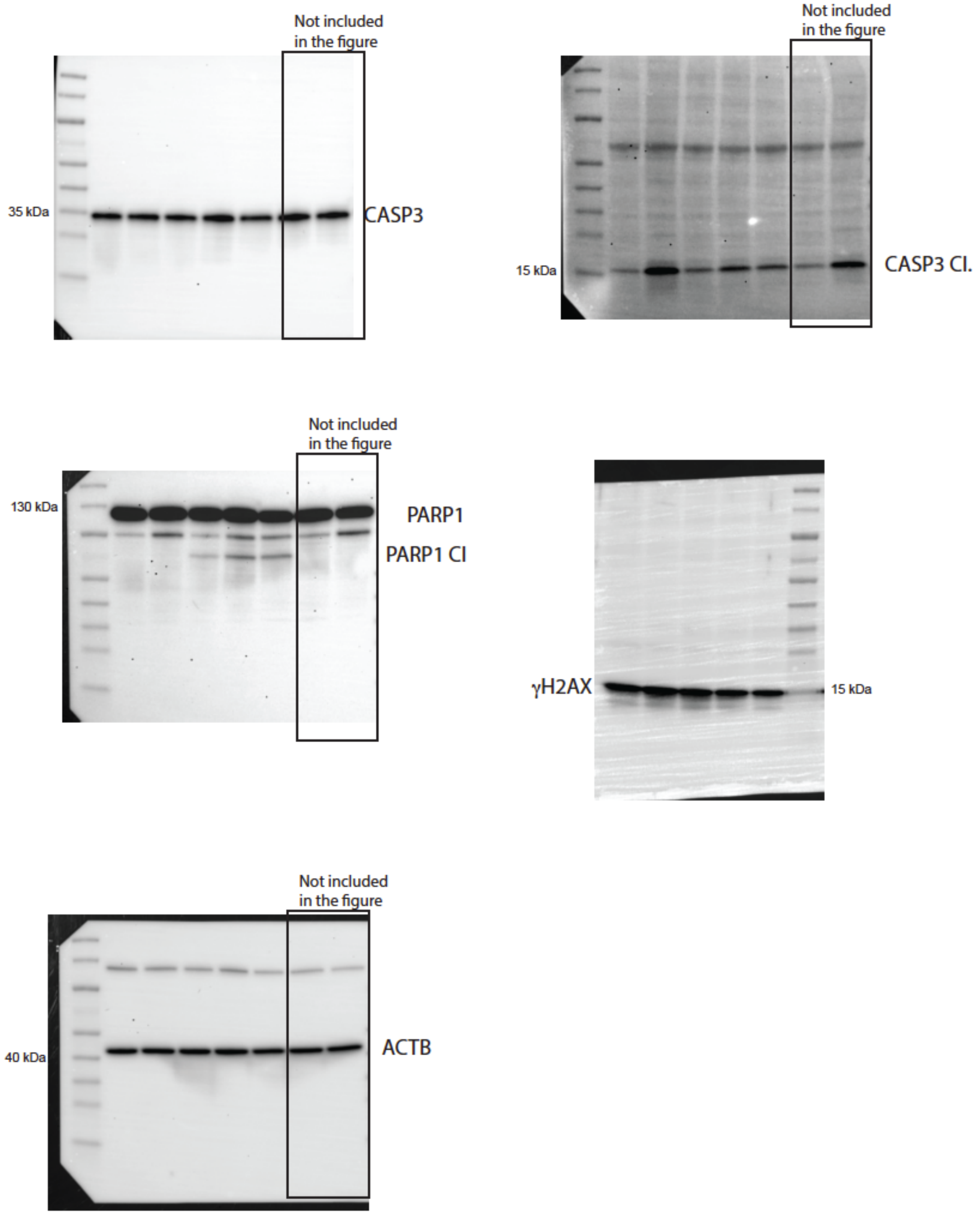

Uncropped western blots from Figure 5E

Figure 5E

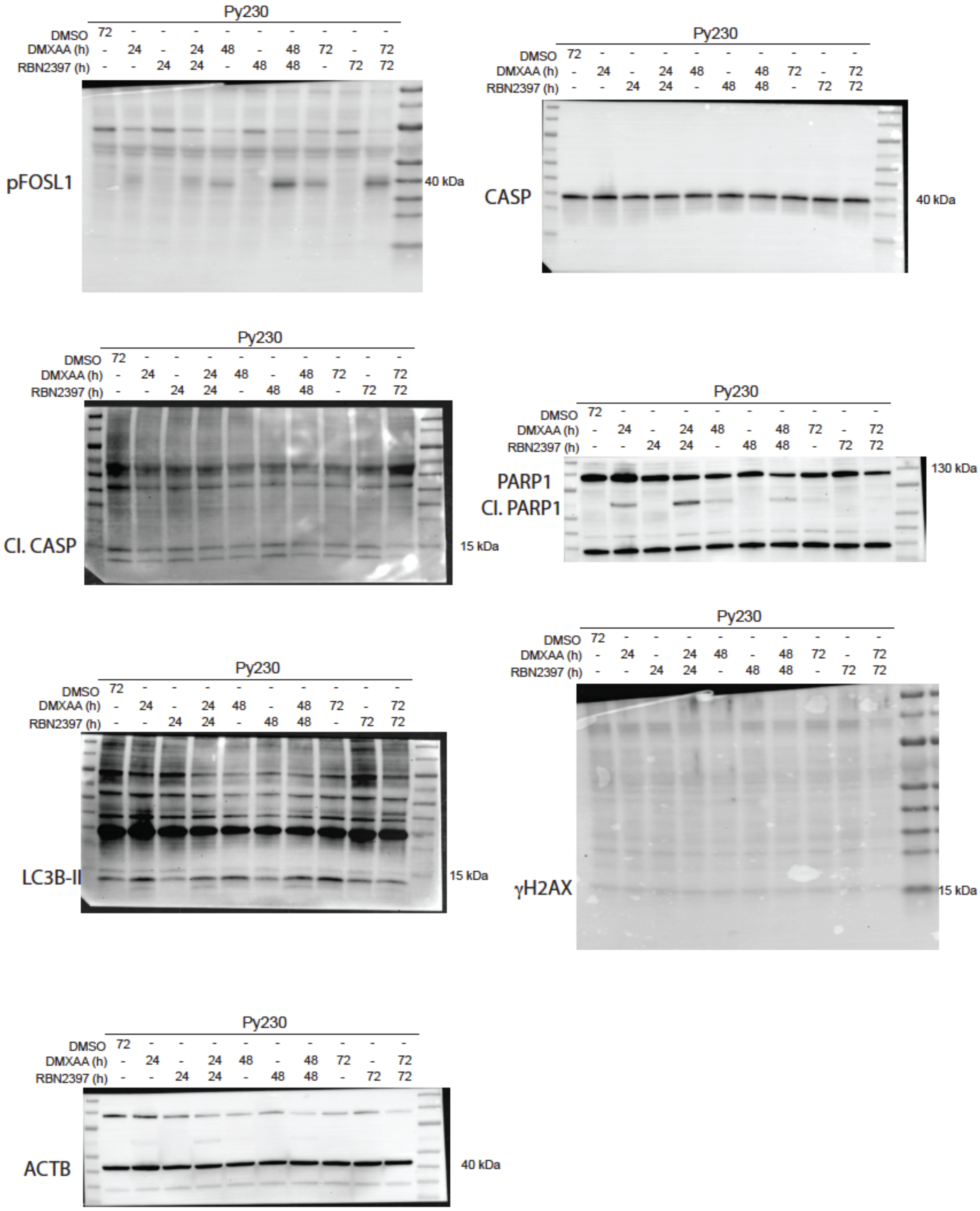

Uncropped western blots from Figure 5I

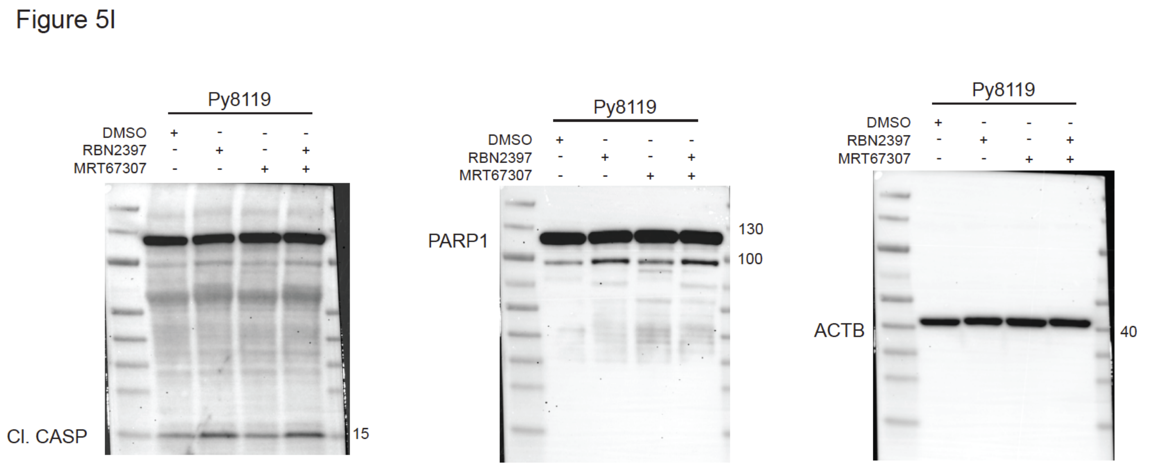

Uncropped western blots from Figure 5K

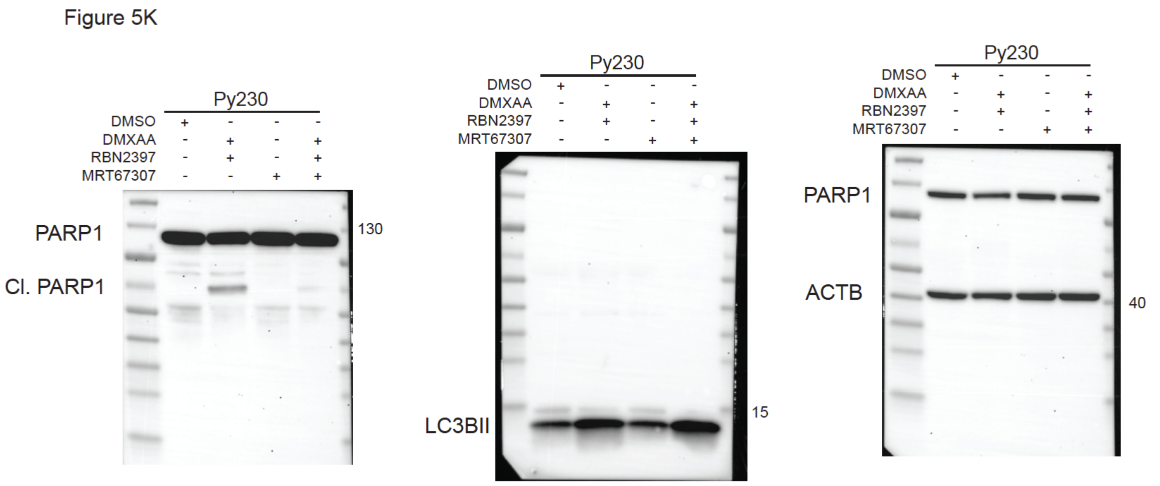

Uncropped western blots from Figure 7A and 7B.

Figure 7A

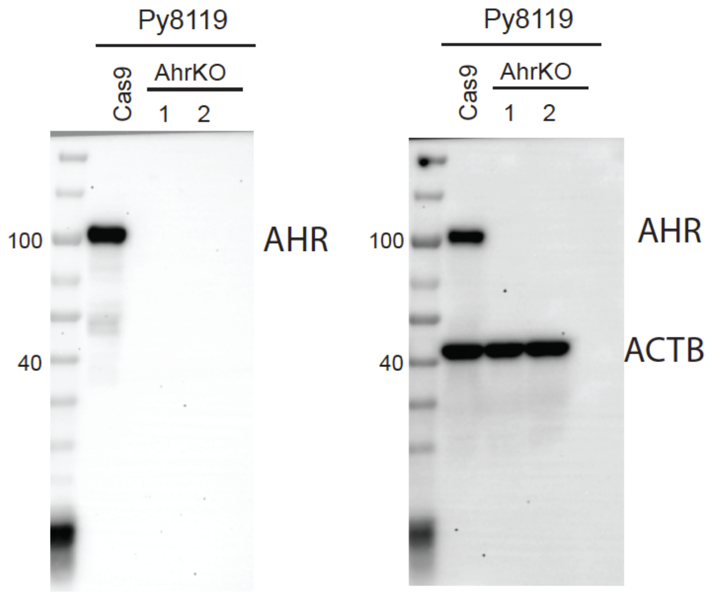

Figure 7B

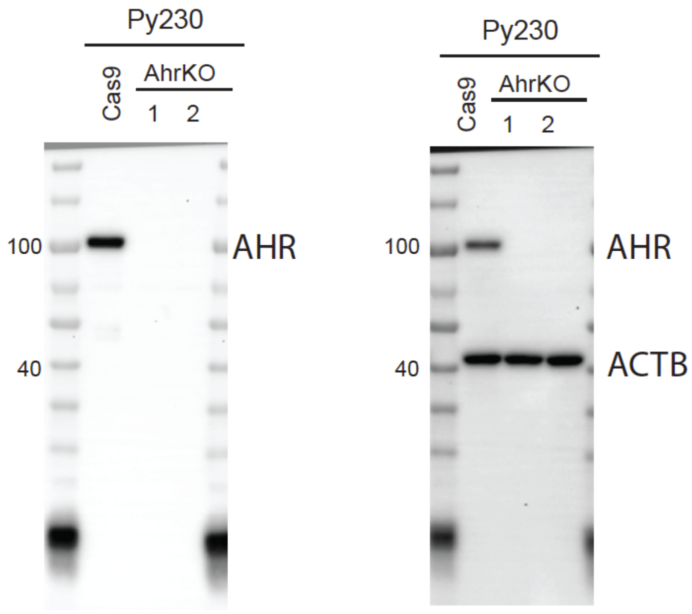

Uncropped western blots from Figure 8.

Figure8

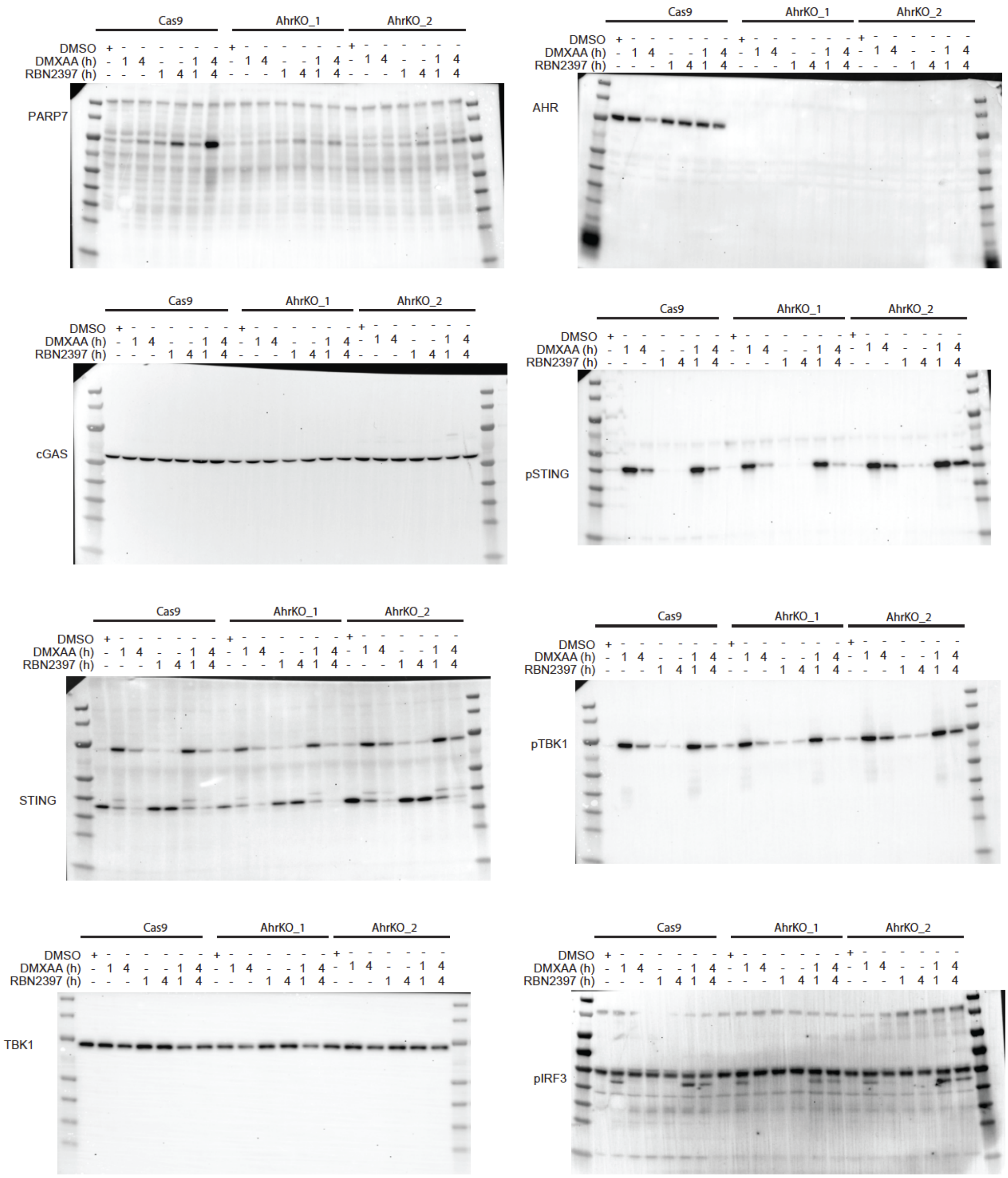

Uncropped western blots from Figure 8 continuted.

Fgure 8

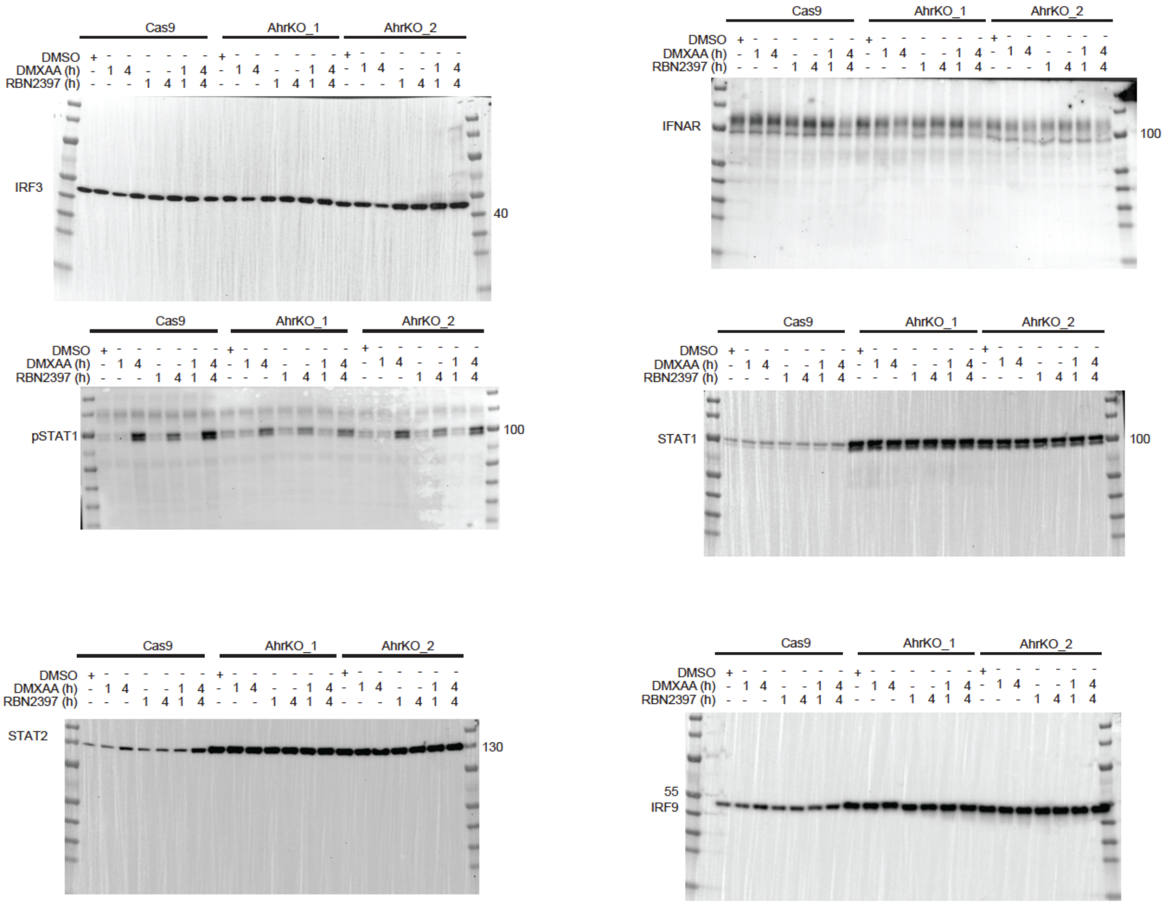

Uncropped western blots from Figure 8 continuted.

Figure 8

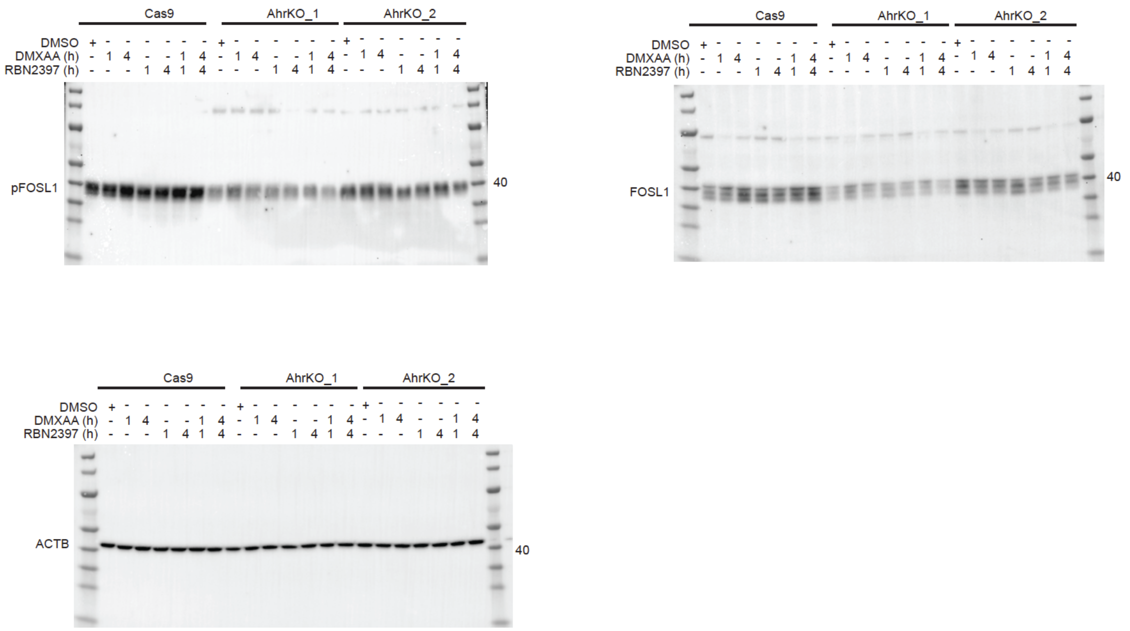

Uncropped western blots from Figure 9K.

Figure 9K

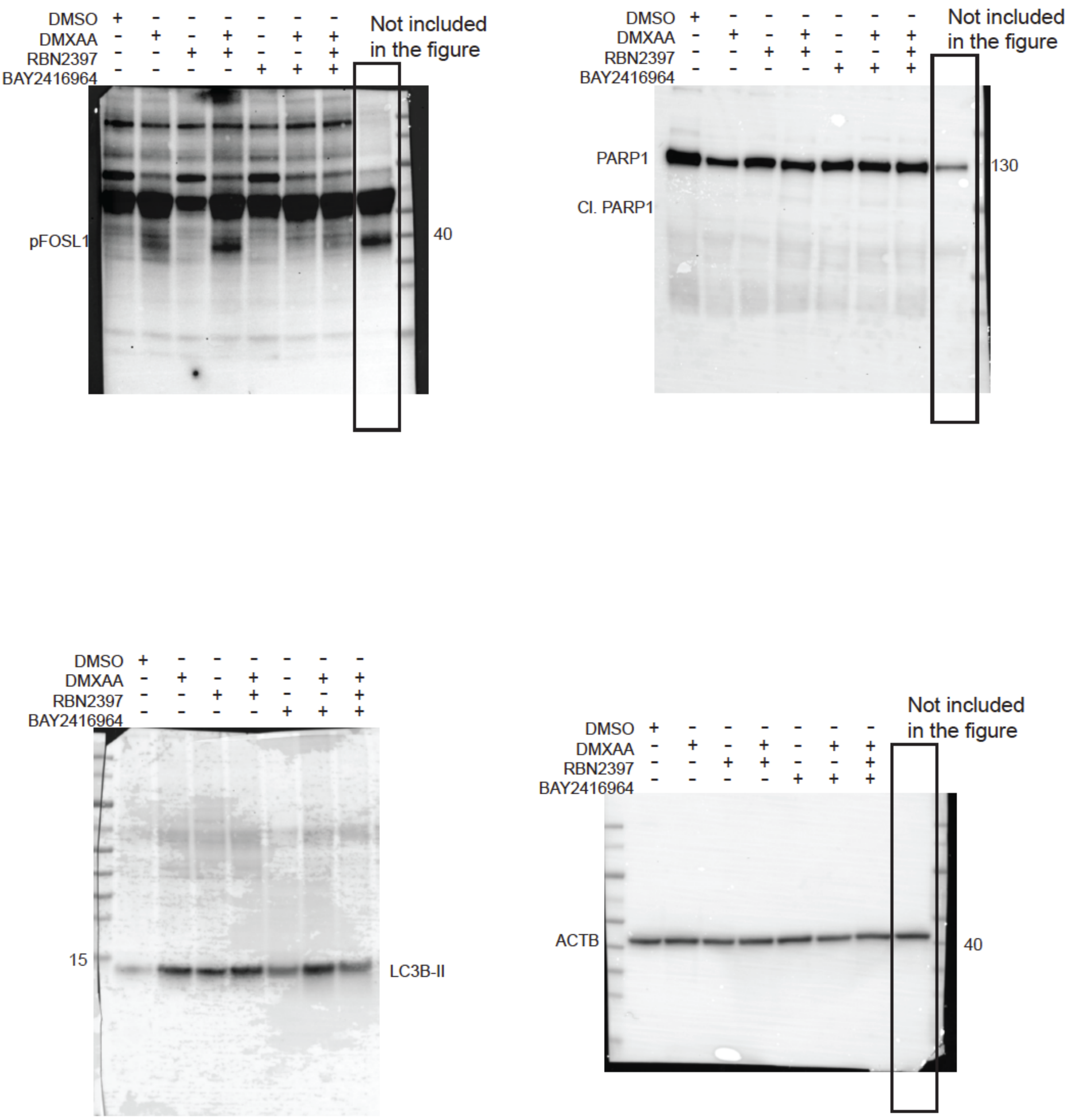

Supplement: Supplementary file 2 — Supplementary Material 2 [file 13402_2025_1150_MOESM2_ESM.pdf]
